# Towards new animal models of pure hypoxic Lance-Adams syndrome: Negative results

**DOI:** 10.1371/journal.pone.0317638

**Published:** 2026-07-08

**Authors:** Geoffroy Vellieux, Delphine Roussel, Anthony Pinto, Nathalie Mougenot, Vincent Navarro

**Affiliations:** 1 Paris Brain Institute – Institut du Cerveau – ICM, Inserm, CNRS, Sorbonne Université, Pitié-Salpêtrière Hospital, Paris, France; 2 AP-HP, EEG Unit, Department of Neurophysiology, Pitié-Salpêtrière Hospital, Paris, France; 3 UMS28, Phénotypage du Petit Animal, Inserm, Sorbonne Université, Paris, France; 4 AP-HP, Epilepsy Unit, Department of Neurology, Reference Center of rare epilepsies, ERN-EpiCare, Pitié-Salpêtrière Hospital, Paris, France; University of Florida, UNITED STATES OF AMERICA

## Abstract

Lance-Adams syndrome is a severe and disabling posthypoxic myoclonus in humans. Its underlying mechanisms are still unknown. We aimed to develop a new animal model of Lance-Adams syndrome. We performed our procedures on Sprague-Dawley rats monitored for basic cardiorespiratory parameters. The Group 1 was sedated and submitted to hypoxia in a dedicated cage where oxygen was replaced with nitrogen. The Groups 2 and 3 were sedated, intubated, ventilated, and submitted to anoxia with the replacement of oxygen by nitrogen in the ventilatory circuit, either continuously (Group 2) or intermittently (Group 3). Rats benefited from cardiopulmonary resuscitation. Each rat was evaluated daily for spontaneous, wandering-induced, and auditory stimuli-induced myoclonic jerks, for the latter using a quantitative myoclonus score. In the Group 1 (n = 19), the duration of hypoxia was 25 ± 20 min for the surviving animals (n = 14/19) and minimal partial pressure of oxygen in the cage was 7.2 ± 0.8%. In the Group 2 (n = 38), the duration of anoxia was 5.6 ± 1.9 min for the surviving animals (n = 23/38). In the Group 3 (n = 30), the total duration of anoxia was 15 ± 3.5 min in the surviving animals (n = 15/30). These three procedures did not allow for generating a myoclonic phenotype. Some refinements of hypoxic/anoxic procedures are still needed to develop an animal model of posthypoxic myoclonus that would be a precious tool to understand better the pathophysiology of Lance-Adams syndrome.

## Introduction

Lance-Adams syndrome (LAS) or chronic posthypoxic myoclonus (PHM) is a severe and disabling neurological condition occurring in human survivors of brain anoxia [1]. It is caused by brain damage resulting from prolonged and deep oxygen deprivation related to various etiologies such as peri-surgery/anesthetic accidents, asthma attacks, myocardial infarction, or intoxication and drug overdoses. The main symptom of LAS is myoclonus, i.e., sudden, brief, and involuntary movements caused by muscular contractions (positive myoclonus) or inhibition of contractions (negative myoclonus). These jerks typically occur during actions and motions, in response to external stimuli such as sounds, or more rarely spontaneously at rest [2,3]. Several anti-myoclonic treatments are available for the patients, including pharmacological anti-seizure medications, such as clonazepam, valproate, and levetiracetam, and neuro-stimulation approaches in medication-refractory patients, such as deep brain stimulation or electroconvulsive therapy [2,4–6]. Physicians caring for patients with LAS face many unmet medical needs. First, LAS is defined only by clinical criteria with no sensitive and specific validated diagnostic biomarkers. Second, the treatments currently available are limited and not effective enough, leaving many patients severely disabled. Third, the underlying pathophysiological mechanisms of LAS are unknown, especially the neuronal generator of myoclonus. We recently showed that myoclonus in patients with LAS originates from the cortex, especially the motor cortex [6]. Identifying the neuroanatomical substrate of myoclonus in LAS may lead to determining a brain target allowing diagnostic and therapeutic advances. An animal model, generated by a mechanism similar to that in humans and mimicking the human phenotype of LAS, would help answer many of these questions. It would particularly participate in elucidating the mechanisms of LAS and facilitate the search for effective therapeutic interventions.

Two animal models of PHM secondary to cardiac arrest (CA) were reported, based on two different types of CA induction. The first used pharmacological induction, in anesthetized, ventilated, and curarized rats, using a transthoracic intracardiac injection of potassium chloride KCl and cessation of ventilation to induce CA [7]. The second one used mechanical induction, in anesthetized and ventilated rats, using an L-shaped loop inserted into the body cavity and a simultaneous external digital chest compression to occlude the major cardiac vessels, including the aorta, ultimately leading to CA [8–10]. However, these two models have certain limitations. First, the authors used ischemia instead of hypoxia/anoxia. Second, the pharmacological induction model displayed a high mortality rate and cardiac and renal morbidity due to the intracardiac injection of KCl. The mechanical induction model was based on an invasive surgical protocol. Finally, myoclonus was assessed only after anoxia (with no comparison before/after anoxia), and only in response to auditory stimuli.

Thus, we aimed to develop a novel animal model of LAS using a simpler and less invasive method, with a lower mortality rate, in a procedure that better matches the human hypoxic mechanism of LAS. Moreover, we aimed to evaluate the animals’ muscular jerks before and after hypoxia spontaneously, during motions, and in response to external stimuli, compared to controls. These different procedures did not allow us to replicate in animals the myoclonic phenotype observed in humans.

## Materials & methods

The experiments complied with the European Union guidelines (Directive 2010/63/EU) and were approved by the local Ethics Committee Charles Darwin CEEACD/N°5 and the French Ministry of Research under APAFIS no. #35162–2022060716206563 and #43666–2023083015044880. The experiments were performed in the animal facilities of the Paris Brain Institute (registration number B751319) and the “Phénotypage du Petit Animal” unit (registration number B751320) on male Sprague Dawley rats (total n = 105, obtained from Charles River Laboratories). After receiving the animals, all rats underwent one week of acclimatization to the housing area (with a 12-hour light/dark cycle, 55 ± 10% humidity, 20–24°C temperature, phonic isolation, free access to food and water, and suitable enrichment in cages) and one week of habituation to human handling. Rats (total n = 105) were aged 6.6 ± 1 weeks and weighed 269 ± 55 g on the day of the anoxia procedure.

We used two approaches to induce a rodent phenotype that approximates the human phenotype of LAS, either in non-intubated or intubated rats.

### Hypoxia in non-intubated rats

**Fig 1:**
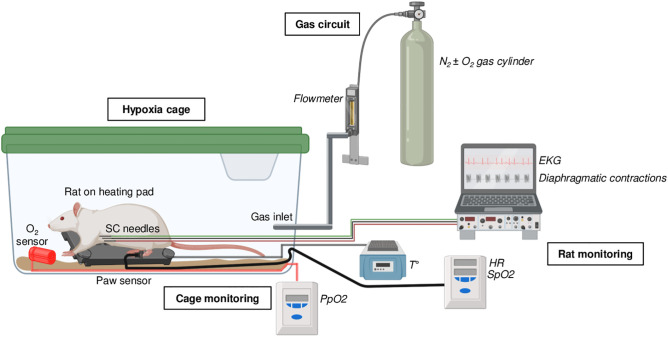
Experimental set-up for hypoxia in sedated non-intubated rats within a dedicated hypoxia cage. EKG: electrocardiogram, HR: heart rate, N_2_: nitrogen, O_2_: oxygen, PpO_2_: partial pressure of O_2_ inside the cage, SC: subcutaneous, SpO_2_: oxygen saturation, T°: temperature.

**Fig 2:**
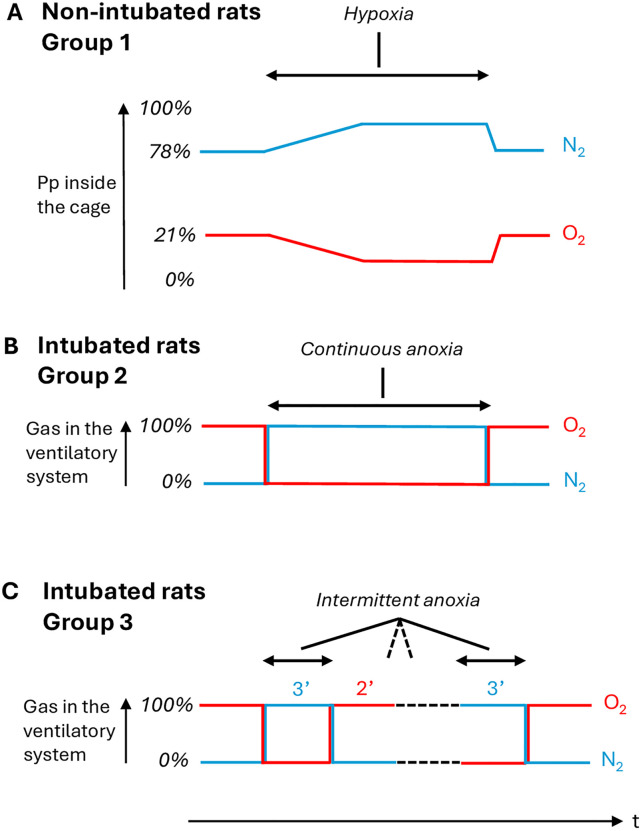
Experimental protocols. (A) Experimental protocol of hypoxia in non-intubated rats. Animals were placed in a dedicated cage. Continuous hypoxia was obtained by replacing the cage’s ambient air (containing 21% O2) with a gas mixture containing mainly (or only) nitrogen and a low (or zero) concentration of oxygen and stopped just before the respiratory arrest. The slope of the decrease and the minimum value of PpO_2_ varied according to the gas cylinder used and the flowmeter settings. (B) and (C) Experimental protocol of anoxia in intubated rats. (B) Continuous anoxia: rats were ventilated with a constant 100% N_2_ ventilation (blue) for various anoxia durations between animals, stopped when the heart rate decreased below 150 beats/min. (C) Intermittent anoxia: rats were ventilated with alternating 3-minute periods of 100% N_2_ ventilation (blue) and 2-minute periods of 100% O_2_ ventilation (red) for various total anoxia durations (sum of each 3-minute period of 100% N_2_ ventilation) between animals, stopped when the heart rate decreased below 150 beats/min. N_2_: nitrogen, O_2_: oxygen, Pp: partial pressure, t: time.

Rats (n = 27) were anesthetized with an intraperitoneal injection of ketamine (75–100 mg/kg) and xylazine (5–10 mg/kg) and protective eye gel was applied. The cardiorespiratory signals of the animals were monitored during the experiments with subcutaneous needles for the electrocardiogram (EKG) and diaphragmatic contractions (Neurosoft), and with a paw sensor for the heart rate (HR) and oxygen saturation (SpO2) (Rat Pulse Oximeter Sensor and MouseSTAT Jr. Pulse Oximeter, EMKA Technologies-Kent Scientific). Rats were placed on a heating pad (Physitemp) inside a *homemade* hypoxia chamber (Tecniplast GM500 cage body and flat top) containing an air oxygen sensor for monitoring the partial pressure of oxygen (PpO_2_) inside the cage (OXY-SEN oxygen monitor, LAR Process Analysers AG-Alpha Omega Instruments). Hypoxia was obtained by replacing the ambient air of the cage with a gas mixture containing a low oxygen concentration for up to one hour. For this purpose, the hypoxia chamber was connected to a gas cylinder (Linde) containing a gas mixture of oxygen (O_2_) and nitrogen (N_2_), with zero or a low concentration of O_2_: 0% (N_2_ 100%), 2% (N_2_ 98%), 4% (N_2_ 96%), or 6% (N_2_ 94%), enabling the depth of hypoxia to be varied. A flowmeter was connected between the gas cylinder and the cage allowing the rate of gas entry into the cage to be varied from 0.5 to 12 l/min (Figs 1 and 2A). These two variable parameters (O_2_/N_2_ ratios in gas cylinders and gas inflow rate with flowmeter) allowed us to vary on one hand the minimal PpO_2_ at equilibrium inside the cage and on the other hand to vary the slope of decrease in the PpO_2_ inside the cage during the procedure. After hypoxia, the cage was opened allowing a rapid return to a normal ambient oxygen concentration (21%), and, in the case of bradycardia or CA, the rats received chest compressions (200/minute) and intraperitoneal epinephrine injections (0.02 mg/kg).

We performed this hypoxia procedure in two subgroups of rats. The first experiments were done for the preliminary evaluation of our hypoxia set-up in a subset of rats (n = 8, pilot group of sedated non-intubated rats) that were not studied with the myoclonus assessment (see below). Another group of rats (n = 19, Group 1) was studied using the daily myoclonus assessment before and after hypoxia (see below).

Group 1 control rats (n = 4) were anesthetized with an intraperitoneal injection of ketamine (75 mg/kg) and xylazine (5 mg/kg), placed on a heating pad, and stayed in ambient air until they recovered from anesthesia, and were studied using the daily myoclonus assessment before and after anesthesia.

### Anoxia in intubated rats

Rats (n = 68) were anesthetized by inhalation of 3% isoflurane and protective eye gel was applied. Rats received analgesia with a subcutaneous meloxicam injection (1.5 mg/kg) and were placed on a heating pad (Physitemp). They were then intubated and ventilated (Harvard Rodent Respirator, ventilatory rate of 45/min, ventilator tidal volume of 1 ml/100 g, O_2_ 100%). Anesthesia was then maintained with 1.5% isoflurane to complete the procedure. The cardiorespiratory signals of the animals were monitored during the experiments with subcutaneous needles for the EKG and HR and with a paw sensor for SpO_2_ (Animal Oximeter Pod, PowerLab 4/26, and FE231 Bio Amp, ADInstruments). Anoxia was obtained by replacing the 100% O_2_ with 100% N_2_ in the ventilatory system, either continuously or intermittently (see below). For the resuscitation after the anoxia period, the rats were again ventilated with continuous 100% O_2_, and the isoflurane maintenance sedation was ceased. In the case of bradycardia or CA, they received chest compressions (200/min) and intraperitoneal epinephrine injections (0.02 mg/kg). As soon as spontaneous ventilation resumed, the rats were progressively weaned from the ventilator and extubated.

We performed two types of anoxia protocols on the intubated rats (Figs 2B and C).

### Continuous anoxia

Rats (n = 38, Group 2) were subjected to continuous anoxia, i.e., constant 100% N_2_ ventilation, for up to 10 minutes (Fig 2B). In a subset of 24/38 rats, we administered an epinephrine injection just before the anoxia procedure and another one at the end of the anoxia regardless of the HR, to increase the duration of anoxia and the survival rate. We stopped the anoxia and proceeded to resuscitation when the HR was < 150 beats/min regardless of the anoxia duration (see results).

### Intermittent anoxia

Rats (n = 30, Group 3) were subjected to intermittent anoxia, i.e., alternating 3-minute periods of 100% N_2_ ventilation and 2-minute periods of 100% O_2_ ventilation, for up to 33 minutes (Fig 2C). We stopped the anoxia and proceeded to resuscitation when the HR was < 150 beats/min regardless of the total anoxia duration (see results).

Groups 2 and 3 control rats (n = 6) were anesthetized by inhalation of 3% isoflurane, received a subcutaneous meloxicam injection (1.5 mg/kg), and were placed on a heating pad (Physitemp). They were intubated and ventilated for 10 minutes with O_2_ 100%, and anesthesia was maintained with 1.5% isoflurane. After 10 minutes, the isoflurane maintenance sedation was ceased, and they rapidly recovered from anesthesia. They were studied with the daily myoclonus assessment before and after anesthesia.

### Monitoring of animals, especially myoclonus assessment

All rats were monitored daily for behavior and main health parameters (locomotion, agitation or immobility, breathing, coat, face, posture, weight, vocalizations) paying close attention to signs of discomfort, stress, or pain. Rats showing pain signs received buprenorphine (0.05 mg/kg subcutaneous). Gel food was given to animals at first signs of motor impairment. The specific criteria used to determine when animals should be euthanized were: (i) weight loss > 20% compared to the first weighing before the hypoxia/anoxia procedure, (ii) shock, lethargy, unconsciousness, immobility, (iii) epileptic seizures lasting more than 10 minutes, and (iv) daily myoclonus score > 200 (see below) on five consecutive days. The animals were then sacrificed during the day by an injection of pentobarbital (140 mg/kg).

The severity of muscular jerks was assessed daily before and after the anoxia. It included two approaches for all rats. The first was a subjective visual analysis with the daily observation of freely moving animals to detect spontaneous and wandering-induced abnormal muscular jerks. The second was an objective quantitative analysis of the rats’ reactions to auditory stimuli to detect stimuli-induced abnormal muscular jerks compared to controls. We adapted the method already described [7]. For this purpose, each rat was daily and individually subjected to a train of 45 identical auditory stimuli (80 dB, 40 ms, 1 Hz). Muscle jerks in response to each sound were scored with a daily myoclonus score as follows: 0 = no reaction; 1 = ear twitch; 2 = ear and head jerk; 3 = ear, hand, and shoulder jerk; 4 = generalized jerk; and 5 = generalized jerk leading to a jump. For each daily assessment, the myoclonus score thus ranged from 0 to 225 for each rat. Rats were evaluated daily from 8–12 days before hypoxia/anoxia to 10–20 days after depending on groups. All rats were euthanized by an injection of pentobarbital (140 mg/kg) at the end of the myoclonus assessment period.

Experimental details for each rat included in this study is provided in S1 Table.

### Statistical analysis

Differences between the characteristics of the groups were performed with GraphPad Prism 10.2.1 and assessed using t-test or Fisher’s exact test when appropriate, and *p* < 0.05 was considered a significant effect. Results are further presented as mean ± standard deviation or as median (interquartile).

To compare the results of the myoclonus score between experimental and controls groups, we used a linear mixed-effect model (LMM) to account for the repeated-measures structure of our data. We fitted the following LMM to our data: score ~ time * group + (1 | rat_id), using “score” as the myoclonus score (primary outcome), “time” as the days before and after hypoxic or anoxic protocol, “group” as the experimental condition controls vs experimental protocol (hypoxia, continuous anoxia or intermittent anoxia), and “(1 | rat_id)” as the random intercept for each animal to account for within-subject correlation.

Analyses were conducted in R (v 4.5.1) using the “lme4” (v 1.1.37), “lmerTest” (v3.1.3), and “emmeans” (v2.0.0) packages. Model convergence was confirmed via diagnostic plots (residuals vs. fitted values, Q-Q plots) and the absence of singularity warnings.

## Results

**Fig 3:**
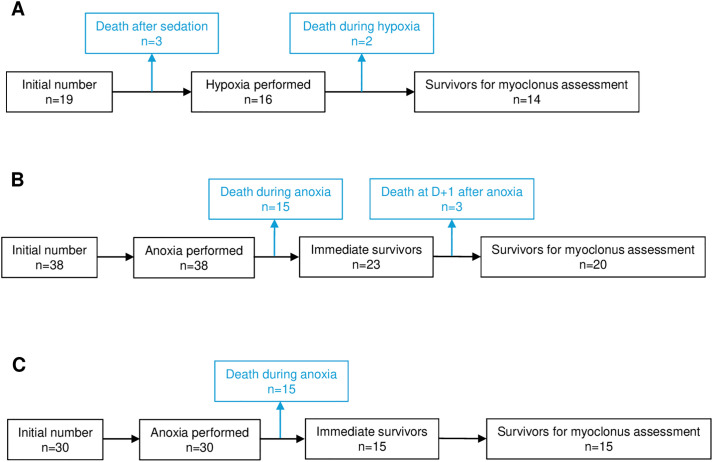
Evolution of animal numbers throughout the various protocols. Animals that died during experiments are highlighted in blue. (A) Hypoxia in sedated non-intubated rats, Group 1. (B) Continuous anoxia in sedated intubated rats, Group 2. (C) Intermittent anoxia in sedated intubated rats, Group 3.

**Fig 4:**
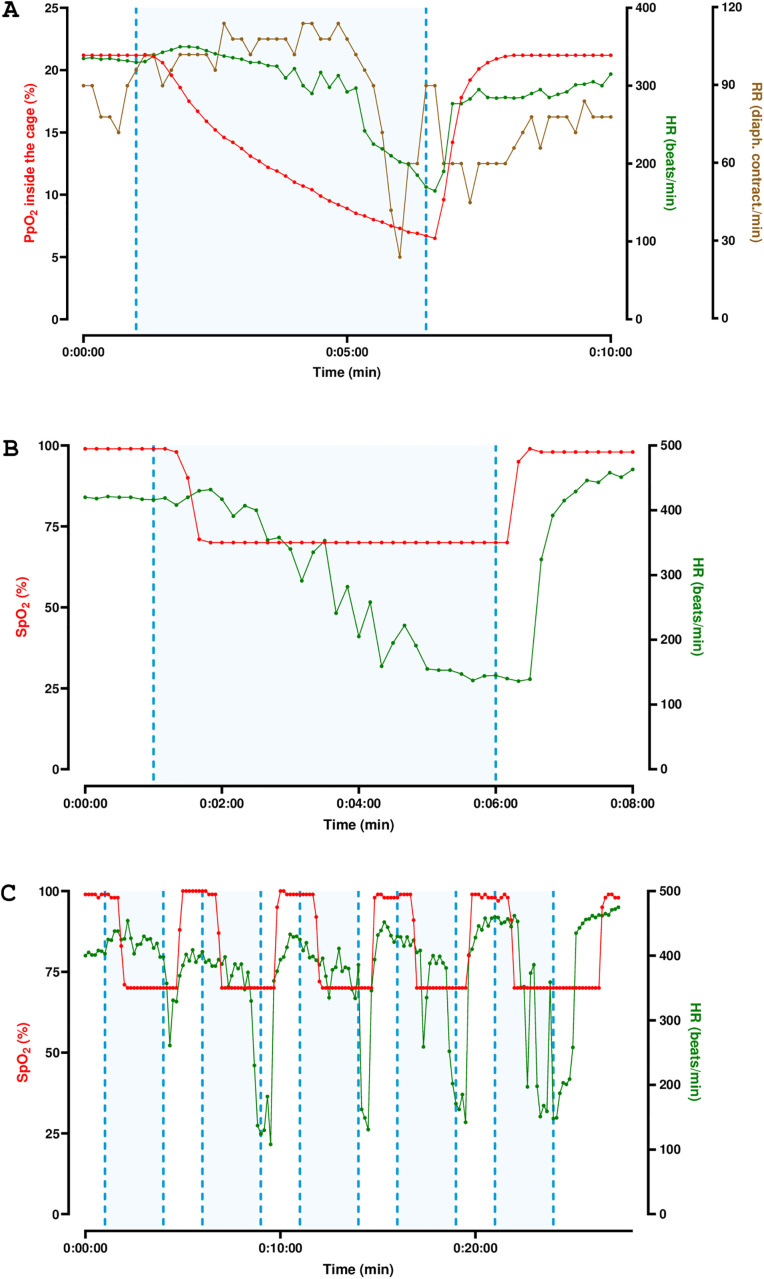
Evolution of cardiorespiratory signals during experiments. (A) Evolution of the partial pressure of oxygen inside the cage (PpO_2_, red), heart rate (HR, beats/min, green), and respiratory rate (RR, diaphragmatic contractions/minute, brown) of a Group 1 sedated non-intubated rat submitted to hypoxia with the gas cylinder containing 100% N_2_ (no O_2_) and flowmeter at 1 l/min. The hypoxic period is highlighted in blue. Hypoxia lasted 5.5 min and was interrupted because of brutal bradypnea near respiratory arrest. (B) and (C) Evolution of the oxygen saturation (SpO_2_, red) and the heart rate (HR, beats/min, green) of a sedated intubated rat submitted to continuous (B, Group 2) or intermittent (C, Group 3) anoxia. In (B) and (C), the paw sensor for SpO_2_ had a lower detection threshold of 70%. (B) The anoxic period is highlighted in blue. Anoxia lasted 5 min and was interrupted because of bradycardia (HR around 140 beats/min). (C) The anoxic periods are highlighted in blue. The total anoxia lasted 15 min (5 periods of 3 min each). In (A), (B), and (C), dots are displayed every 10 seconds.

**Fig 5:**
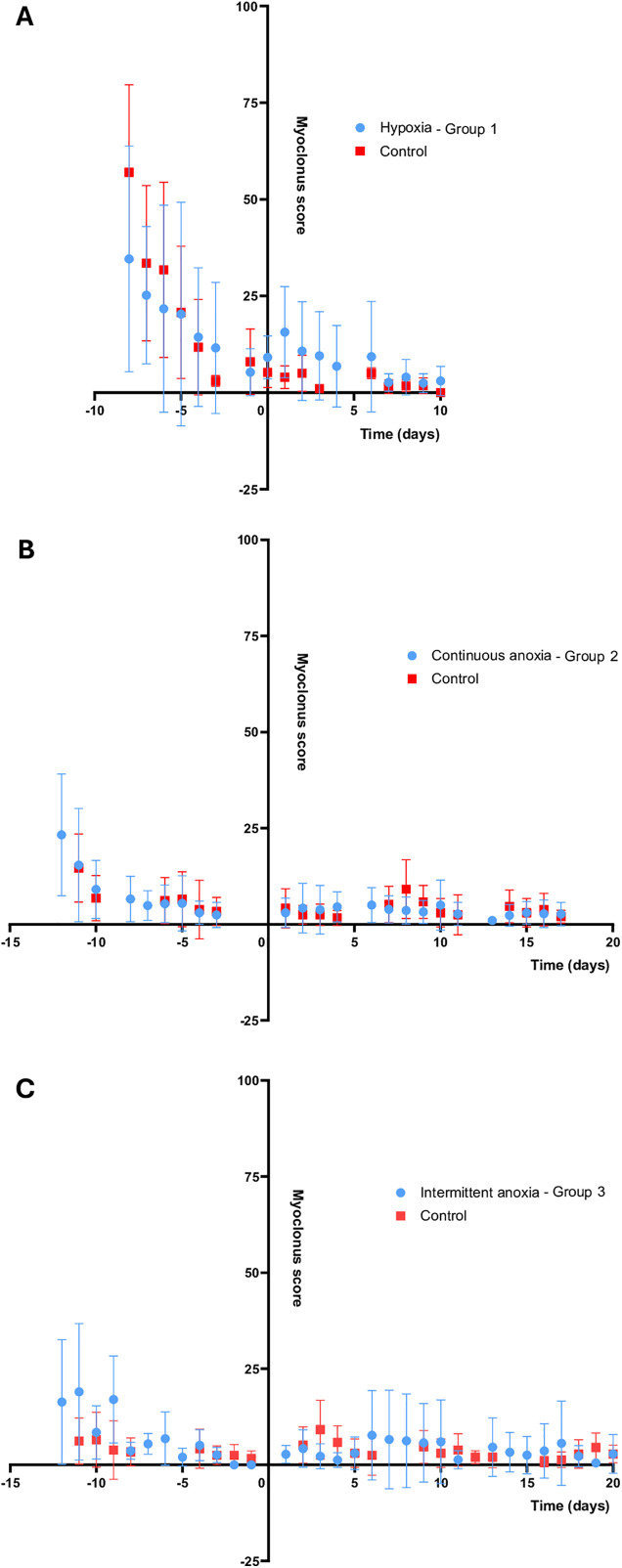
Evolution of the auditory stimuli-induced myoclonus scores. (A) Evolution of the auditory stimuli-induced myoclonus score for the Group 1 non-intubated rats evaluated daily from 8 days before hypoxia to 10 days after. Hypoxia occurred on day 0. Each point represents the daily median (± interquartile) of the group: blue for Group 1 hypoxic rats (n = 14/19 surviving animals) and red for controls (n = 4). No difference was observed between the groups. (B) and (C) Evolution of the auditory stimuli-induced myoclonus score for the intubated rats (Groups 2 and 3). (B) Rats subjected to continuous anoxia (Group 2) were evaluated daily from 12 days before anoxia to 17 days after. Anoxia occurred on day 0. Each point represents the daily median (± interquartile) of the group: blue for Group 2 anoxic rats (n = 20/38 surviving animals) and red for controls (n = 6). No difference was observed between the groups. (C) Rats subjected to intermittent anoxia (Group 3) were evaluated daily from 12 days before anoxia to 20 days after. Anoxia occurred on day 0. Each point represents the daily median (± interquartile) of the group: blue for Group 3 anoxic rats (n = 15/30 surviving animals) and red for controls (n = 6). No difference was observed between the groups. For the three experiments (A), (B), and (C), we only observed variable acoustic startle reflex during the first days of exposition to auditory stimuli which rapidly decreased due to long-term habituation. The daily myoclonus score for each rat extends from 0 (45x0) to 225 (45x5). For the sake of clarity, the Y axis is interrupted at 100.

Our first approach was to induce hypoxia in sedated non-intubated rats placed in a hypoxia chamber (Table 1). During the first experiments on the pilot group of sedated non-intubated rats (n = 8, results not shown), animals died when they reached respiratory arrest (defined as the cessation of visible respiratory movements and diaphragmatic contractions), despite a return to ambient air (O_2_ 21%), chest compressions, and multiple epinephrine injections during resuscitation. We thus decided to stop hypoxia just before the respiratory arrest, regardless of the duration of hypoxia, HR, and PpO_2_ level inside the cage. In the Group 1 (n = 19), three rats died just after the sedation with an intraperitoneal injection of ketamine/xylazine (100/10 mg/kg) and were not submitted to the hypoxia procedure, explaining why we reduced the dosage of ketamine/xylazine to 75/5 mg/kg to reduce mortality. Two rats died during the hypoxia procedure despite resuscitation. For the 14/19 (74%) surviving rats, the duration of hypoxia was 25 ± 20 min and the minimal PpO_2_ at equilibrium inside the cage was 7.2 ± 0.8% (Figs 3A and 4A). We did not observe visible spontaneous and wandering-induced abnormal muscular jerks in freely moving Group 1 post-hypoxic rats up to 10 days after the hypoxia procedure. For the comparison of the daily auditory stimuli-induced myoclonus scores in the Group 1 compared to Group 1 controls, the LMM output is summarized in Table 2. We found a significant main effect on time (p < 0.001), suggesting a significant modification of the myoclonus score over time. The statistical model did not show significant global difference between the Group 1 and Group 1 controls (Fig 5A). We also did not demonstrate any significant interaction between the time and the Group 1.

**Table 1:** Experimental procedures and Groups. bpm: beats per minute, CA: cardiac arrest, HR: heart rate, K: ketamine, min: minutes, PpO_2_: partial pressure of oxygen, SD: standard deviation, sec: seconds, X: xylazine.

Rat cohorts (n)	Experimental method	Anesthesia procedure	O_2_ concentration	Threshold for resuscitation	Epinephrin injections (0.02 mg/kg i.p.)	Survivors - n - Hypo/anoxia duration (mean ± SD; range: min – max) - Minimum O_2_ (mean ± SD; range: min – max)	Non-survivors - n - Hypo/anoxia duration (mean ± SD; range: min – max) - Minimum O_2_ (mean ± SD; range: min – max)
Pilot group of non-intubated rats (8)	Continuous hypoxia in our *homemade* hypoxia chamber	K + X 100/10 mg/kg	0% (n = 4) 2% (n = 1) 4% (n = 0) 6% (n = 3)	At respiratory arrest	Multiple doses during resuscitation	0	− 8 − 161 ± 56 sec - Minimal PpO_2_ concentration inside the cage: 7.9 ± 2%
Aborted Group 1 experiments (3)	None	K + X 100/10 mg/kg	None	None	None	0	3 (died just after the sedatives injection)
Group 1 (11)	Continuous hypoxia in our *homemade* hypoxia chamber	K + X 75/5 mg/kg	0% (n = 7) 2% (n = 4) 4% (n = 5) 6% (n = 0)	Just before respiratory arrest	Multiple doses during resuscitation of CA (n = 2)	− 14 (all studied with the daily myoclonus assessment) − 25 ± 20 min; 5.5–60 min - Minimal PpO_2_ concentration inside the cage: 7.2 ± 0.8%; 5.1–8.2%	− 2 − 47 min; 42–52 min - Minimal PpO_2_ concentration inside the cage: 6.4%; 5.1–7.6%
Group 1 controls (4)	Continuous ambient air in chamber	K + X 75/5 mg/kg	Ambient air	None	None	− 4 (all studied with the daily myoclonus assessment) - Until spontaneous wake-up - PpO_2_ inside the cage: 21%	0
Group 2 (38)	Continuous anoxia by intubation and ventilation with 100% N_2_	Isoflurane, 3% induction and 1.5% maintenance	0%	HR < 150 bpm	One dose before the procedure (n = 24), then multiple doses during resuscitation if needed	− 23 (20/23 studied with the daily myoclonus assessment) − 5.6 ± 1.9 min; 2–10.5 min − 0% O_2_ during the whole anoxia experiment	− 15 − 6.7 ± 1.3 min; 4.8–10 min − 0% O_2_ during the whole anoxia experiment
Group 3 (30)	Intermittent anoxia by intubation and ventilation with 100% N_2_	Isoflurane, 3% induction and 1.5% maintenance	Alternating 3-min periods with 100% N_2_ and 2-min periods with 100% O_2_	HR < 150 bpm	Multiple doses during resuscitation if needed	− 15 (all studied with the daily myoclonus assessment) − 15 ± 3.5 min; 10.5–21 min − 0% O_2_ during the repeated bouts of 100% N_2_ ventilation	− 15 − 12.9 ± 4.0 min; 5–21 min − 0% O_2_ during the repeated bouts of 100% N_2_ ventilation
Groups 2 and 3 controls (6)	Continuous O_2_ exposure by intubation and ventilation with 100% O_2_	Isoflurane, 3% induction and 1.5% maintenance	100%	None	None	− 6 (all studied with the daily myoclonus assessment) - Until spontaneous wake-up - O_2_ inside the ventilatory circuit: 100%	0

**Table 2:** LMM output for comparison of the Group 1 vs Group 1 controls.

Effect	Estimate (SE)	95% CI	df	p-value	Sig.
Time	−1.8833 (0.3188)	[-2.5118, -1.2547]	1	<0.001	***
Group 1	0.2689 (5.3754)	[-11.1293, 11.6672]	1	0.9607	ns
Time:group	0.523 (0.3659)	[-0.1982, 1.2443]	1	0.1543	ns

Our second approach was to induce anoxia in sedated intubated rats (n = 68). During the first experiments of continuous anoxia, we observed that 75% of rats died when the HR was < 150 beats/min despite a return to 100% O_2_ ventilation, chest compressions, and multiple epinephrine injections during resuscitation. We thus decided to stop the anoxia when the HR decreased to a 150 beats/min threshold, regardless of the duration of the anoxia. In the Group 2 (n = 38, Table 1), the overall mean duration of anoxia was 6 ± 1.8 min, and the immediate survival rate was 61% as 15 animals died during the anoxia procedure despite resuscitation. The systematic injections of epinephrine (n = 24/38) just before and after the anoxia procedure (regardless of the HR) did not allow for a significant increase in the duration of anoxia compared to rats that did not receive it (n = 14/38) (6.3 ± 1.6 min vs 5.5 ± 2 min (*p* = 0.22)) and immediate survival rate (67% (8/24 animals died) vs 50% (7/14 animals died) (*p* = 0.49)). For the 23/38 (61%) surviving rats, the mean duration of anoxia was 5.6 ± 1.9 min. Three animals that recovered from anoxia and anesthesia (after an anoxic period of 5, 10, and 10.5 min each) had immediate difficulties in moving and feeding and died the day after the anoxia despite attentive care (Figs 3B and 4B). In the 20 remaining rats, we did not observe visible spontaneous and wandering-induced abnormal muscular jerks in freely moving Group 2 post-anoxic rats up to 17 days after the anoxia procedure. For the comparison of the daily auditory stimuli-induced myoclonus scores in the Group 2 rats compared to Groups 2 and 3 controls, the LMM output is summarized in Table 3. We found a significant main effect of time (p < 0.001), suggesting a significant modification of the myoclonus score over time. The statistical model did not show significant global difference between the Group 2 and Groups 2 and 3 controls (Fig 5B). We also did not demonstrate any significant interaction between the time and the Group 2.

**Table 3:** LMM output for comparison of the Group 2 vs Groups 2 and 3 controls.

Effect	Estimate (SE)	95% CI	df	p-value	Sig.
Time	−0.1795 (0.065)	[-0.3073, -0.0517]	1	<0.001	***
Group 2	0.0729 (1.4842)	[-2.9862, 3.132]	1	0.9612	ns
Time:group	−0.1323 (0.0772)	[-0.2841, 0.0195]	1	0.0874	ns

In the Group 3 (n = 30, Table 1), the survival rate was 50% as 15/30 animals died during the anoxia procedure despite resuscitation. For the 15 surviving rats, the total duration of anoxia, i.e., the sum of periods with 100% N_2_ ventilation, was 15 ± 3.5 min (Figs 3C and 4C). We did not observe visible spontaneous and wandering-induced abnormal muscular jerks in freely moving Group 3 post-anoxic rats up to 20 days after the anoxia procedure. For the comparison of the daily auditory stimuli-induced myoclonus scores in the Group 3 compared to Group 2 and 3 controls, the LMM output is summarized in Table 4. We found a significant main effect of time (p < 0.001), suggesting a significant modification of the myoclonus score over time. The statistical model did not show significant global difference between the Group 3 and Groups 2 and 3 controls (Fig 5C). We also did not demonstrate any significant interaction between the time and the Group 3.

**Table 4:** LMM output for comparison of the Group 3 vs Groups 2 and 3 controls.

Effect	Estimate (SE)	95% CI	df	p-value	Sig.
Time	−0.0789 (0.0536)	[-0.1843, 0.0265]	1	<0.001	***
Group 3	1.7031 (2.1795)	[-2.8483, 6.2546]	1	0.4439	ns
Time:group	−0.098 (0.0656)	[-0.227, 0.031]	1	0.1360	ns

## Discussion

Lance-Adams syndrome is an extremely disabling neurological disorder occurring in patients who survived from anoxia-ischemia. Although this condition is rare, it is probably underdiagnosed. The mortality after anoxia-ischemia is unfortunately still very high. For example, the overall survival rate of out-of-hospital CA is 8% in a recent international, prospective, multicenter study [11]. However, the care chain following CA and the cardiopulmonary resuscitation techniques have improved over the last few decades. There will be more survivors in the years to come, most of them with post-anoxic complications such as hypoxic-ischemic brain injury [12]. Among these post-anoxic brain complications, the prevalence of Lance-Adams syndrome, which is currently unknown, will undoubtedly increase.

We need to understand better the pathophysiological mechanisms of LAS which remain largely unknown to improve the severe neurological disabilities associated with LAS. An animal model will be of great value in identifying the neuroanatomical substrates underlying LAS and helping develop effective curative and preventive treatments. Two rodent models of LAS have been reported, using ischemic procedures. We aimed to create a novel rodent model of LAS with an alternative protocol specifically using pure hypoxia/anoxia instead of ischemia. We failed to elicit a myoclonic phenotype using hypoxia-anoxia procedures in non-ventilated or ventilated rodents.

The two rodent models of LAS employed either pharmacological or mechanical ischemic procedures on male Sprague-Dawley rats weighing 250–300 g [10]. These animals showed similar behavioral and pharmacological features as seen in patients with LAS: auditory stimuli-induced myoclonic jerks during the days after CA that were reduced by some antimyoclonic drugs, which are partially effective in humans. However, they are both based on global cerebral ischemia due to CA rather than primitive hypoxia (leading or not to hypoxic CA), which limits its construct validity. In contrast, two thirds of patients with LAS present with a primary respiratory hypoxic event: respiratory arrest/failure without cardiac arrest in 24% of patients and respiratory arrest/failure followed by CA in 41% of patients [2]. Second, the mortality and cardiac and renal morbidity are high in the pharmacological induction model, whose rate-limiting factors are successful resuscitation and postoperative survival due to cardiac and renal failure. Third, the mechanical induction model needs an invasive and cumbersome surgical procedure, using the mechanical obstruction of major cardiac vessels with a loop inserted into the body cavity for 7 to 10.5 min to induce PHM in animals while limiting the mortality rate [13]. Finally, myoclonus was reported in these studies only in response to auditory stimuli after the anoxia, which limits its phenotypic and predictive validity. The intensity of stimuli-induced myoclonic jerks peaked on day 4 and 14 after CA on the myoclonus score for the mechanical and pharmacological induction model respectively, and then gradually declined to the initial basal level in a few weeks. However, with the mechanical induction model, the intensity of audiogenic myoclonic jerks was more severe at day 2 after CA using capacitive sensors and polymyographic recordings [14]. In contrast, the main activation mode of myoclonus in humans suffering from LAS is action and myoclonus is particularly intense during movements [2]. Nevertheless, rats which underwent CA with mechanical induction showed also ataxic movements during weeks following anoxia perhaps due to action-induced myoclonic jerks [14].

To refine the elaboration of valid PHM animal models, our goal was to develop a novel rodent model of LAS with an alternative protocol specifically using pure hypoxia/anoxia instead of ischemia. Patients with LAS indeed mainly suffered from a primary hypoxic respiratory event [2]. The biochemical and cellular mechanisms involved in neuronal damage caused by hypoxia/anoxia and ischemia may vary [15]. This variability may lead to heterogeneous biochemical consequences in the micro-environment of neurons, for example, on extracellular glutamate concentrations [16], gene expression [16,17], or neuropathological changes [18,19]. One critical question raised by our negative findings is why ischemic–anoxic cardiac arrest produces chronic startle myoclonus in rodents, whereas nearly fatal pure anoxia as we designed does not. Such difference may be related to the distinct effects of these insults on cortical microcircuit balance, particularly within the motor cortex. Global ischemia induces simultaneous deprivation of oxygen and glucose, leading to rapid ATP depletion, glutamate accumulation, and sustained depolarization. Experimental studies indicate that GABAergic interneurons—especially parvalbumin-positive fast-spiking cells—are highly vulnerable to oxygen–glucose deprivation and exhibit impaired functional recovery [20]. In contrast, surviving pyramidal neurons may display relative hyperexcitability during reperfusion. This imbalance favors cortical disinhibition. Such selective inhibitory dysfunction within motor cortex circuits could create a hyperexcitable state capable of generating stimulus-sensitive myoclonus. In addition, reperfusion-related oxidative stress, inflammatory activation, and blood–brain barrier disruption may promote maladaptive plasticity within cortico-thalamo-cortical and cortico-cerebellar loops, further destabilizing motor output networks, in the context of a possible involvement of subcortical structures in posthypoxic myoclonus [13,14]. By contrast, in pure anoxia with preserved perfusion, glucose delivery and metabolic clearance remain intact. Severe hypoxia rapidly suppresses neuronal firing, but the metabolic collapse may be less abrupt than in ischemia. Interneuronal adaptive mechanisms may be relatively preserved, and the absence of reperfusion injury may limit excitotoxic amplification and long-term network reorganization. Rather than producing selective disinhibition, pure middle anoxia may induce depression of cortical activity insufficient to trigger chronic hyperexcitable motor circuits. More severe anoxia, requiring cardio-circulatory assistance, may nevertheless cause some specific neuronal damages.

We chose the rat species for our experiments as the standardized myoclonus score was developed for rats [7]. Rats and humans have similar hemodynamic parameters and rat anatomy and physiology are well known, facilitating the extrapolation of findings to humans. We chose male Sprague-Dawley rats because they were previously employed for LAS models and CA rodent models [21]. Young adult rats around 250–300 g were easy to handle, especially during intubation. This choice is debatable and may influence oxygen tolerance and subsequent pathophysiological responses. However, our experiments were fully exploratory through a pilot study, only based on previous successful ischemic procedures of other teams. We thus took advantage of these successful procedures to reuse the same rat strain, age, and sex. Fundamental differences in species might explain our failure. The human neocortex has undergone such expansion and reorganization that many human brain regions have no direct homologues in rodents. The smooth lissencephalic cortex of the rat differs markedly from the folded gyrencephalic cortex of humans and primates, which may impact the capacity of the rodent brain to exhibit complex network phenomena such as sustained involuntary movements after global hypoxic injury. These gyrification differences correlate with quantitative and qualitative distinctions in cortex organization. Non-human primates and humans exhibit a larger proportion of isocortex and expanded prefrontal regions than rodents, which are associated with higher-order motor control, complex sensory integration, and sustained cognitive processing not paralleled in rodent models [22]. Post-hypoxic myoclonus in humans, as a gyrencephalic species, may likely engage distributed cortical loops involving extensive sensorimotor territories, including motor, premotor and supplementary motor areas that are disproportionately expanded in gyrencephalic cortices [6]. The absence of sulci and extensive gyri in rats might limit the emergence or expression of certain network-level disturbances that underlie sustained myoclonic syndromes after diffuse hypoxic injury in humans [23]. However, some authors have reported myoclonic phenotypes in rodents through various contexts, for example as already specified post-hypoxic myoclonus [10], but also progressive myoclonic epilepsy [24] or juvenile myoclonic epilepsy [25,26], outlining the fact that it seems realistic to try to create a myoclonic phenotype in rodents.

We first aimed to induce a LAS phenotype in a hypoxia chamber in sedated non-intubated rats. However, we faced two main issues. We could not use an inhalation sedation method with hypnotic gas because our homemade hypoxia chamber was not equipped with a closed gas circuit with a filtration system to collect the released gas. We therefore used an intraperitoneal injection with hypnotic drugs. Whereas the combination of ketamine and xylazine is widely used in anesthesia for laboratory animals with some advantages, some negative effects such as cardiac depression, bradycardia, hypotension, and respiratory depression have been reported [27,28]. This may have contributed to the early death of some of our animals. Xylazine should be avoided in future experiments with anoxia. Second, we did not have any efficient ventilatory support when rats presented with hypoxic bradypnea and then respiratory arrest. Thus, we could not sustain deep and prolonged oxygen deprivation. However, due to the partial (not total) oxygen deprivation, we maintained animals in this hypoxic condition for an average of 25 min. We then modified the procedure to produce a LAS phenotype in sedated and intubated rats to sustain deep and prolonged oxygen deprivation, either with continuous or intermittent anoxia. In this second group of animals, we took advantage of the animals’ ventilation circuit to use gas inhalation for sedation, especially isoflurane, which presents a moderate cardio-respiratory depression compared to xylazine. Gas anesthesia is usually safe because the sedation level can easily be controlled during maintenance, and anesthesia risks are usually very low [29]. In most cardiorespiratory arrest animal models, anesthesia in small animals such as rodents is carried out using narcotic gases such as isoflurane [21]. Using this procedure, we were able to induce profound anoxia, without the risk of respiratory arrest. Prolonged anoxia finally induced cardiac arrest, which was not avoided by adrenergic injections. This latter procedure of anoxia, stopped when the bradycardia worsens, also failed to generate spontaneous, wandering-induced, or auditory stimuli-induced myoclonic jerks compared to controls. Moreover, the mortality rates of our procedures were high, ranging from 26% in non-intubated Group 1 rats to 39 and 50% in intubated Group 2 continuous and Group 3 intermittent anoxia rats. However, these high mortality rates are unfortunately consistent with the clinical reality of severe anoxia patients. It is particularly instructive to note that our pure anoxia procedures resulted in high mortality, indicating that the manipulations were sufficiently severe to provoke serious pathophysiology, while generating no myoclonic phenotype.

Moreover, post-anoxic resuscitation itself, including reoxygenation and mechanical ventilation parameters, may contribute to secondary brain injury and should therefore be considered in the pathophysiological interpretation of post-anoxic phenomena. Experimental and clinical studies have shown that hyperoxia following return of spontaneous circulation can exacerbate oxidative stress and neuroinflammation. In animal models of cardiac arrest, ventilation with 100% oxygen after resuscitation has been associated with worse neurological outcomes compared with normoxic strategies [30]. More recent experimental work has also demonstrated increased inflammatory and apoptotic signaling in the brain following hyperoxic resuscitation [31]. In parallel, clinical data suggest that extreme PaCO₂ values, particularly severe hypocapnia, are associated with poorer neurological outcomes after cardiac arrest [32,33]. These findings support the concept that post-resuscitation management may modulate the extent of global cerebral injury through oxidative and cerebrovascular mechanisms. Importantly, the current literature does not identify reoxygenation toxicity per se as a specific mechanistic trigger of post-hypoxic myoclonus. While oxidative stress and inflammation may contribute to the overall severity of neuronal damage, available evidence does not support a direct and selective role of hyperoxia or ventilator-induced effects in generating myoclonic activity. Rather, these factors appear to modulate injury severity at a global level.

Our protocols of hypoxia/anoxia were not sufficiently prolonged to induce a myoclonic phenotype in surviving animals. Human patients who develop Lance-Adams syndrome receive prolonged intensive care with mechanical ventilation and hemodynamic monitoring. Some animals that died in our models might have developed myoclonus if we had implemented prolonged ventilation or post-hypoxic intensive care, which our current protocols did not allow. Conversely, cardiocirculatory arrest induced by more prolonged anoxia resulted in significant mortality. These manipulations could therefore be refined by adding a capnograph and intravascular arterial and venous catheters to intubated animals. It would allow us to perform capnography-guided procedures, invasively monitor arterial blood pressure, and administer catecholamines, such as epinephrine and norepinephrine, needed for animals’ resuscitation. Our experimental procedures highlight thus the importance of invasively monitoring arterial blood pressure. This would allow us to assess the dynamics of blood pressure changes in our model and to examine the correlation between the kinetics of blood pressure decline, mortality, and the phenotype of surviving animals. However, these procedures are complex and need a specialized and trained team. This would improve the survival rate during these manipulations, while at the same time increasing the duration of anoxia to optimize the chances of inducing a myoclonic phenotype when the animals wake up. We must define an experimental window in which anoxia is sufficient to induce neuronal injury and a myoclonic phenotype, while avoiding an anoxic insult of excessive severity, with high mortality or excessively severe sequelae that would prevent proper evaluation of the animals’ phenotype. Several experimental parameters must therefore be considered: for instance, duration, severity, and continuous/intermittent design of anoxia, changes in carbon dioxide levels, effects of post-anoxia ventilation.

While our attempts to develop a pure hypoxic model of Lance-Adams syndrome were unsuccessful, these negative findings provide crucial guidance for the scientific community. The failure to induce myoclonus despite severe anoxic injury underscores the importance of ischemic components and species-specific brain architecture in disease pathophysiology. By documenting these unsuccessful approaches, we prevent redundant research efforts and encourage development of more sophisticated models that better reflect human clinical conditions, ultimately advancing our understanding of this debilitating neurological disorder.

Once this post-hypoxic myoclonus animal model has been developed, it can be explored in many ways, including immunohistochemical analyses and electrophysiological *in vivo* recordings. This could provide a wealth of highly accurate data for understanding the pathophysiology of Lance-Adams syndrome in humans.

## Supporting information

S1 Table Experimental details for each rat included in this study. The age, weight, type of the anoxic procedure, the duration of anoxia, the injection of epinephrin before and after anoxia, the post-anoxia outcome, the values of the daily myoclonus score are indicated for each rat.
(XLSX)
