# Supplementary material for: Towards new animal models of pure hypoxic Lance-Adams syndrome: Negative results
Source: PLoS One. 2026 Jul 8;21(7):e0317638. doi: 10.1371/journal.pone.0317638 (PMC13345465; doi:10.1371/journal.pone.0317638)
Supplement: Attachment — Submitted filename: Comments on myoclonus paper.pdf [file pone.0317638.s005.pdf]

I continue to support publishing this manuscript for the reasons I stated in my prior review, which remain in full effect and won't be described further. The authors responded to my prior comments by editing the manuscript and it is greatly improved in places. However, I believe that it must undergo further editing before publication, so that the experimental procedures and results are transparent to investigators that may want to improve upon this study. The suggested edits below are not meant to indicate reduced enthusiasm for the study, but rather are meant to enhance its organization, precision, and language in order to enhance its impact. I acknowledge that the awkward use of English at times may be due to the authors being native French writers.

1. Reorganize and clearly state the experimental groups to be consistent with the revised Abstract.

The authors must restructure their experimental groups to be consistent with the Abstract. Currently, it is very difficult to piece together how the various groups differed from one another along the many dimensions in which they varied, as this information is highly fragmented among the Methods, Results, and Figure Legends. Obviously, it is essential to structure the experimental groups identically in the Abstract and the body of the paper.

To be helpful, I took the liberty of assembling a Table of Experimental Procedures and Groups based on what was written. I very strongly suggest that the authors include this Table in the main body of the paper and restructure their Methods and Results sections to match the Table (edit and include missing information highlighted in yellow). Please edit all other manuscript sections to be organized with regard to this this table. Also, reduce redundancy. As examples, the Discussion may need to be edited to match the experimental groups in certain places; the figures may need to be edited to reflect those groups; information about independent variables in each of the groups may need to be recalculated and moved from the Results to the Methods sections (i.e., mean anoxia duration, etc.). Note: I recognize that some of the information in the Table is in Figure 3, but I find Figure 3 to fall significantly short of the experimental detail that needs to be fully transparent in order to achieve the real purpose of this paper which is to alert investigators not to reproduce methods that do not produce a PHM phenotype in rats.

It is important to emphasize that there are 3 experimental groups and 2 control groups in the myoclonus experiment, with total n=59 for the myoclonus experiments from a total of 105 rats.

Table of Experimental Procedures and Groups (n=105 rats, n=59 for myoclonus testing)

Data presented as mean +/- SD or SEM (state which one)

Rat Cohorts	n	Experimental Method	Anesthesia	Threshold for resuscitation	O_2 concentration	Number of Survivors/Non-survivors	Epinephrine	Survivors<br><br>Hypoxia or anoxia duration; minimum cage PO_2	Non-Survivors<br><br>Hypoxia or anoxia duration; minimum cage PO_2	n for myoclonus testing
Pilot Study	8	Continuous hypoxia in hypoxia cage	Ketamine/Xylazine 75-100/5-10 mg/kg<br><br>[Note: since this occurred before the Aborted Experiments and Group 1, wasn't this consistently 100/10? If so, state that dose; if not state the number of rats at each dose].	At respiratory arrest	State	0/8	Multiple 0.02 mg/kg i.p. doses post-hypoxia during resuscitation		Average:<br><br>Range:<br><br>xx to 60 min	0
Aborted Experiments	3	None	Ketamine/Xylazine 100/10 mg/kg			0/3				0
Group 1	16	Continuous hypoxia in hypoxia cage	Ketamine/Xylazine 75/5 mg/kg	≤ 140-150 bpm	Variable over 0, 2, 4, & 6%<br><br>State number of rats at each O_2 concentration	14/2	Multiple 0.02 mg/kg i.p. doses post-hypoxia if bradycardia (n=x) or cardiac arrest (n=x)	Average duration: 25 +/- 20 min<br><br>Range duration:<br><br>Average PO_2: 7.2 +/- 0.8%<br><br>Range PO_2:<br><br>This is confusing since some rats may have had > 0% O_2	Average duration:<br><br>Range duration:<br><br>Average PO_2:<br><br>Range PO_2:<br><br>This is confusing since some rats may have had > 0% O_2	14
Group 1 Control	4	Ambient air	Ketamine/Xylazine 75/5 mg/kg			4/0	None			4
Group 2	38	Continuous anoxia by intubation & ventilation with 100% N_2	Isoflurane 3% induction/1.5% maintenance	≤ 140-150 bpm	0%	20/18	One 0.02 mg/kg i.p., dose before anoxia; multiple doses after anoxia; n=24	Average : 5.6 +/- 1.9 min<br><br>Range:	Average :<br><br>Range:	20
Group 3	30	Intermittent anoxia by intubation & ventilation with 100% N_2	Isoflurane 3% induction/1.5% maintenance	≤ 140-150 bpm	Repeated bouts of 3 min 100% N_2 followed by 2 min 100% O_2	15/15	State as above, if none, state None	Average:<br><br>15 +/- 3.5 min<br><br>Range: xx to 33 min	Average :<br><br>Range:	15
Groups 2 and 3 Control	6	100% O_2, by intubation and ventilation for 10 min	Isoflurane 3% induction/1.5% maintenance		100%	4/0	None			6

2. Comparison to prior studies of mechanical whole-brain ischemia

It is no surprise that rats with strong hearts perhaps exceeding 60 bpm through a bout of hypoxia or anoxia did not exhibit PHM. In our experiments with the mechanical approach, PHM never resulted if perfusion was not reduced to near zero in a few seconds and maintained there for at least 7 min with anoxia and near 0 mmHg blood pressure (shock) for that duration. Note, in perhaps all of our PHM cases, a greatly weakened heart was itself already reduced to near 0 bpm by 5 min of mechanical ischemia that was sustained for the subsequent duration of anoxia (7-10 min total) before resuscitation.

Also in our and others' experience, which the authors could have deduced from carefully reading the mechanical ischemia PHM literature, short-acting ketamine anesthesia was used alone - without xylazine, because the addition of xylazine as a long-duration CNS depressant and as an adjuvant to prolong ketamine's action invariably prevented surviving prolonged anoxia/ischemia (100% mortality).

Thus, in the present experiments, the investigator's use of xylazine explains the 100% mortality in the Pilot Study (see Table) in which the threshold for resuscitation was severe (complete and sustained respiratory suppression). The subsequent use of a far less pathological threshold (140-150 bpm) as a reaction to the Pilot Study mortality (even ensured by pre-anoxia epinephrine in Group 2!), would certainly prevent the PHM phenotype in any rat surviving those procedures. It can be inferred, therefore, that Group 1-3 non-survivors did not die of the pathophysiology that produces PHM, but of something different (but see below re: the variable threshold). Inserting the requested data in the new Table (yellow highlights, averages and ranges) will help readers understand whether other factors may have contributed (i.e., greater hypoxia/anoxia duration, lesser cage PO_2, etc.).

3. Resuscitation threshold.

Regarding the heart rate threshold for resuscitating Groups 1-3, the authors should describe this precisely. The threshold is described as "below 140-150 bpm," which is vague. How far below (surely not 0 bpm, but 10?, 100?, 145?)? Why describe the threshold as a range? Do the authors, instead, mean that the threshold was between 140 and 149 bpm? Nearly that? The threshold should be clarified and edited everywhere it is described. How variable were the minimum heart rates across rats? Add threshold data to the Table if you have it (average and range across rats for survivors and non-survivors), as this may be clarifying (perhaps non-survivors had lower heart rates) as help to future investigators. As a placeholder, the new Table shows ≤ 140-150 bpm, but that needs to be edited too.

4. Control groups.

It is remarkably incorrect to write that previous studies of rat startle PHM did not use pre-anoxia comparisons or comparisons to control animals (lines 79-81). The authors should carefully read Truong et al (2000) *Movement Disorders,* p. 26, Welsh et al (2002) *Myoclonus and Paroxysmal Dyskinesias, Advances in Neurology*, p. 307, and Welsh et al (2002) *ibid*, p. 331 in which both controls were used. Remarkably, the authors cited Truong et al (2000), indicating they did not read it. And, I am concerned that the authors did not read the two Welsh et al (2002) papers which were provided in my previous review. The authors should carefully read all 3 of those papers, revise their text based on their reading and citing of them, and consider them more carefully with regard to Point 2 and in consideration of their mechanistic explanations in the Discussion. By scholarly reading those papers and others that followed them, the authors will find yet more instances of the use of appropriate control groups, if they care to.

5. Non-standardized oxygen concentration (0, 2, 4, and 6%) in Group 1 (lines 116-118).

The authors should explain how this was handled in their experimental design and specify it in the new Table. It is unclear why oxygen was varied and when the authors chose to do this. Please explain. Introducing this as an unaccounted-for variable is concerning. How did that relate to mortality?

Language and Other Edits

6. The phrase “rats benefitted from the myoclonus assessment” (or its converse “rats that did not benefit from the myoclonus assessment”) used throughout the manuscript is incorrect and must be changed everywhere “benefitted” is used (note: no rat benefitted from any aspect of these experiments; all experimenters benefitted). I suggest the phrase “rats that were studied using the myoclonus assessment” or some proper English variant thereof.

7. The phrase “systematically died” is awkward and morbid. According to what system did they die, and what evidence was collected to reveal that system? I suggest removing the adverb “systematically,” unless the authors are trying to make a physiological point in which case it should be explained. As for all suggested edits, this should be applied globally throughout the manuscript.

8. Line 166: “At the first signs of awakening, the rats were progressively weaned from the ventilator and extubated.” In my experience, weaning from the ventilator begins when spontaneous breaths first begin to fight the ventilator. That is not synonymous with awakening, as most rats are in coma at this stage (see the literature). In my experience, some rats post severe anoxia will begin to breathe spontaneously and can be weaned from the ventilator, but never awaken. Clarify.

9. Line 172: “We systematically administered an epinephrine injection…” Remove the adverb “systematically,” or describe your “system” if it is something more important than filling a syringe.

10. Line 178: why not say “intermittent hypoxia,” since that is the defining term of this section?

11. Line 183: Begin paragraph with “Control rats (n=6) were…” or “To serve as no-hypoxia controls for Groups 2 and 3,…”

12. Line 246: “After the hypoxia procedure”: How long after the hypoxia procedure?

13. Line 317: new paragraph when discussing Group 3. In general, good scientific writing begins the results of each experimental group as a new paragraph.

14. Line 318: restating mortality is unnecessary.

15. I don’t know if this is a journal issue or an author issue, but it is highly distracting and prevents easy reading to have the figure captions inserted into the body of the document without spacing and separated far from the figure. Why aren’t the legends with the figures?

16. Figures: All figures should clearly refer to Groups 1, 2 or 3.

Discussion

17. Line 370: replace “8 to 9.5 min” to “7 to 10.5 min.” Read and cite Welsh et al (2002), *Adv Neurol*, Chapter 32 to see quantitative description of this.

18. Line 373-375. The intensity of the myoclonic jerk in the mechanical cardiac arrest model of PHM does not peak at day 4 post surgery. The best quantitative measures of the PHM audiogenic jerk by capacitive sensors and multi-muscle EMG indicate that it is most severe 2 days post anoxia/ischemia, and decreases nearly linearly over the following 3 weeks. Read and cite Welsh et al (2002), *Adv Neurol*, Chapter 31 to see high-res, quantitative analyses that were designed to be more sensitive than the myoclonus score.

19. Lines 376-377. Rats having undergone mechanical cardiac arrest leading to PHM are often very ataxic for weeks following anoxia, perhaps due to action-induced myoclonic jerks. Although that was not well studied, those rats show slower and jerkier voluntary (i.e. conditioned) arm movements during the period that they show audiogenic myoclonus (see and cite Welsh et al (2002) *Adv Neurol*, Chapter 31). To a degree, those behavioral observations counter the authors’ suggestion here that the mechanical cardiac arrest model lacks face validity to human PHM because it induces audiogenic jerks only. This should be stated.

20. Line 389. See Welsh et al (2002) *Adv Neurol* Chapter 32 and a growing clinical literature about cerebellar involvement in PHM as a complement to M1 involvement. EMG data suggests that rat cardiac arrest PHM is driven by a brainstem generator (see Welsh et al Adv Neurol Chapter 31).

21. Line 441. Replace “note” with “not.”

22. The Discussion is greatly improved from the previous version and appreciated.
