# Supplementary material for: Towards new animal models of pure hypoxic Lance-Adams syndrome: Negative results
Source: PLoS One. 2026 Jul 8;21(7):e0317638. doi: 10.1371/journal.pone.0317638 (PMC13345465; doi:10.1371/journal.pone.0317638)
Supplement: Attachment — Submitted filename: Response to Reviewers-final.docx [file pone.0317638.s007.docx]

**You will find in this document our responses (in blue) to the Reviewers’ comments (in black).**

**Reviewer #4**

The authors have addressed all of my suggestions very well. The results are a useful contribution to the literature. Figure 5 is OK, but still might benefit from larger fonts on axis labels, and suppressing the negative part of the y-axis (since the score can never be negative). I also would have nudged one of the groups to the left or right so the error bars do not overlap. This is purely an artistic suggestion.

We thank the reviewer for this overall comment about the new version of our manuscript.

We could not modify the figure 5 due to a problem with our previous software.

**Reviewer #5**

I continue to support publishing this manuscript for the reasons I stated in my prior review, which remain in full effect and won’t be described further. The authors responded to my prior comments by editing the manuscript and it is greatly improved in places. However, I believe that it must undergo further editing before publication, so that the experimental procedures and results are transparent to investigators that may want to improve upon this study. The suggested edits below are not meant to indicate reduced enthusiasm for the study, but rather are meant to enhance its organization, precision, and language in order to enhance its impact. I acknowledge that the awkward use of English at times may be due to the authors being native French writers.

1. Reorganize and clearly state the experimental groups to be consistent with the revised Abstract.

The authors must restructure their experimental groups to be consistent with the Abstract. Currently, it is very difficult to piece together how the various groups differed from one another along the many dimensions in which they varied, as this information is highly fragmented among the Methods, Results, and Figure Legends. Obviously, it is essential to structure the experimental groups identically in the Abstract and the body of the paper.

To be helpful, I took the liberty of assembling a Table of Experimental Procedures and Groups based on what was written. I very strongly suggest that the authors include this Table in the main body of the paper and restructure their Methods and Results sections to match the Table (edit and include missing information highlighted in yellow). Please edit all other manuscript sections to be organized with regard to this this table. Also, reduce redundancy. As examples, the Discussion may need to be edited to match the experimental groups in certain places; the figures may need to be edited to reflect those groups; information about independent variables in each of the groups may need to be recalculated and moved from the Results to the Methods sections (i.e., mean anoxia duration, etc.). Note: I recognize that some of the information in the Table is in Figure 3, but I find Figure 3 to fall significantly short of the experimental detail that needs to be fully transparent in order to achieve the real purpose of this paper which is to alert investigators not to reproduce methods that do not produce a PHM phenotype in rats.

It is important to emphasize that there are 3 experimental groups and 2 control groups in the myoclonus experiment, with total n=59 for the myoclonus experiments from a total of 105 rats.

As requested by the reviewer, we created a Table 1 with all our experimental results and added it to the new version of our manuscript.

We also renamed throughout the manuscript the experimental groups as requested, i.e. Group 1, Group 2, Group 3, Group 1 controls, and Groups 2 and 3 controls.

2. Comparison to prior studies of mechanical whole-brain ischemia

It is no surprise that rats with strong hearts perhaps exceeding 60 bpm through a bout of hypoxia or anoxia did not exhibit PHM. In our experiments with the mechanical approach, PHM never resulted if perfusion was not reduced to near zero in a few seconds and maintained there for at least 7 min with anoxia and near 0 mmHg blood pressure (shock) for that duration. Note, in perhaps all of our PHM cases, a greatly weakened heart was itself already reduced to near 0 bpm by 5 min of mechanical ischemia that was sustained for the subsequent duration of anoxia (7-10 min total) before resuscitation.

Also in our and others’ experience, which the authors could have deduced from carefully reading the mechanical ischemia PHM literature, short-acting ketamine anesthesia was used alone - without xylazine, because the addition of xylazine as a long-duration CNS depressant and as an adjuvant to prolong ketamine’s action invariably prevented surviving prolonged anoxia/ischemia (100% mortality).

Thus, in the present experiments, the investigator’s use of xylazine explains the 100% mortality in the Pilot Study (see Table) in which the threshold for resuscitation was severe (complete and sustained respiratory suppression). The subsequent use of a far less pathological threshold (140-150 bpm) as a reaction to the Pilot Study mortality (even ensured by pre-anoxia epinephrine in Group 2!), would certainly prevent the PHM phenotype in any rat surviving those procedures. It can be inferred, therefore, that Group 1-3 non-survivors did not die of the pathophysiology that produces PHM, but of something different (but see below re: the variable threshold). Inserting the requested data in the new Table (yellow highlights, averages and ranges) will help readers understand whether other factors may have contributed (i.e., greater hypoxia/anoxia duration, lesser cage PO2, etc.).

We agree that all of the available sedative drugs, used alone or in combination, have side effects. However, the combination of ketamine and xylazine, as a classical anesthetic procedure, was requested by ethical committees and animal facilities. By consequence, this combination may lead to increased mortality, an issue we faced during our experiments. We have now added this important point to the Discussion, emphasizing that xylazine should be avoided in future experiments.

Moreover, as requested by the reviewer, we added all the experimental data in the new Table 1.

3. Resuscitation threshold.

Regarding the heart rate threshold for resuscitating Groups 1-3, the authors should describe this precisely. The threshold is described as “below 140-150 bpm,” which is vague. How far below (surely not 0 bpm, but 10?, 100?, 145?)? Why describe the threshold as a range? Do the authors, instead, mean that the threshold was between 140 and 149 bpm? Nearly that? The threshold should be clarified and edited everywhere it is described. How variable were the minimum heart rates across rats? Add threshold data to the Table if you have it (average and range across rats for survivors and non-survivors), as this may be clarifying (perhaps non-survivors had lower heart rates) as help to future investigators. As a placeholder, the new Table shows ≤ 140-150 bpm, but that needs to be edited too.

To clarify, we specified throughout the manuscript that procedures were stopped when heart rate was lower to 150 bpm.

4. Control groups.

It is remarkably incorrect to write that previous studies of rat startle PHM did not use pre-anoxia comparisons or comparisons to control animals (lines 79-81). The authors should carefully read Truong et al (2000) *Movement Disorders,* p. 26, Welsh et al (2002) *Myoclonus and Paroxysmal Dyskinesias, Advances in Neurology*, p. 307, and Welsh et al (2002) *ibid*, p. 331 in which both controls were used. Remarkably, the authors cited Truong et al (2000), indicating they did not read it. And, I am concerned that the authors did not read the two Welsh et al (2002) papers which were provided in my previous review. The authors should carefully read all 3 of those papers, revise their text based on their reading and citing of them, and consider them more carefully with regard to Point 2 and in consideration of their mechanistic explanations in the Discussion. By scholarly reading those papers and others that followed them, the authors will find yet more instances of the use of appropriate control groups, if they care to.

We are sorry for these errors. We corrected our manuscript and deleted the fact that previous animal models were performed without comparison to control animals.

5. Non-standardized oxygen concentration (0, 2, 4, and 6%) in Group 1 (lines 116-118).

The authors should explain how this was handled in their experimental design and specify it in the new Table. It is unclear why oxygen was varied and when the authors chose to do this. Please explain. Introducing this as an unaccounted-for variable is concerning. How did that relate to mortality?

With procedures on sedated non-intubated rats subjected to hypoxia in a dedicated cage (Group 1), we aimed to create different experimental conditions to explore the parameters that could generate the desired myoclonic phenotype. To this end, we introduced variability into our experimental conditions by using, on the one hand, oxygen/nitrogen cylinders with different concentration ratios (mainly to vary the minimal partial pressure of oxygen at equilibrium during the procedure) and, on the other hand, a flowmeter to vary the gas inflow rate into the hypoxia chamber (mainly to vary the slope of decrease in the partial pressure of oxygen inside the cage during the procedure).

We specified these arguments in our manuscript and the number of rats that received ventilation in the hypoxia chamber with cylinders containing 0%, 2%, 4%, or 6% oxygen in Table 1.

Language and Other Edits

6. The phrase “rats benefitted from the myoclonus assessment” (or its converse “rats that did not benefit from the myoclonus assessment”) used throughout the manuscript is incorrect and must be changed everywhere “benefitted” is used (note: no rat benefitted from any aspect of these experiments; all experimenters benefitted). I suggest the phrase “rats that were studied using the myoclonus assessment” or some proper English variant thereof.

We changed the sentences as requested, from “benefited from…” to “were studied with…”.

7. The phrase “systematically died” is awkward and morbid. According to what system did they die, and what evidence was collected to reveal that system? I suggest removing the adverb “systematically,” unless the authors are trying to make a physiological point in which case it should be explained. As for all suggested edits, this should be applied globally throughout the manuscript.

We removed the word “systematically” as requested.

8. Line 166: “At the first signs of awakening, the rats were progressively weaned from the ventilator and extubated.” In my experience, weaning from the ventilator begins when spontaneous breaths first begin to fight the ventilator. That is not synonymous with awakening, as most rats are in coma at this stage (see the literature). In my experience, some rats post severe anoxia will begin to breathe spontaneously and can be weaned from the ventilator, but never awaken. Clarify.

We totally agree with the reviewer and clarified the sentence as follows: “As soon as spontaneous ventilation resumed, the rats…”.

9. Line 172: “We systematically administered an epinephrine injection…” Remove the adverb “systematically,” or describe your “system” if it is something more important than filling a syringe.

We removed the word “systematically” as requested.

10. Line 178: why not say “intermittent hypoxia,” since that is the defining term of this section?

We replaced the word “discontinuous” to “intermittent” as requested.

11. Line 183: Begin paragraph with “Control rats (n=6) were…” or “To serve as no-hypoxia controls for Groups 2 and 3,…”

We changed the sentence as requested.

12. Line 246: “After the hypoxia procedure”: How long after the hypoxia procedure?

We clarified the sentences throughout the manuscript as follows: “We did not observe visible spontaneous and wandering-induced abnormal muscular jerks in freely moving post-hypoxic/anoxic rats up to X days after the hypoxia/anoxia procedure.”

13. Line 317: new paragraph when discussing Group 3. In general, good scientific writing begins the results of each experimental group as a new paragraph.

We added a new paragraph as requested.

14. Line 318: restating mortality is unnecessary.

As this mortality rate is specific for this group, we chose to precise this data.

15. I don’t know if this is a journal issue or an author issue, but it is highly distracting and prevents easy reading to have the figure captions inserted into the body of the document without spacing and separated far from the figure. Why aren’t the legends with the figures?

We totally agree with the reviewer, but as requested by the Instructions for authors of PLOSOne (https://journals.plos.org/plosone/s/submission-guidelines): “***Figure captions must be inserted in the text of the manuscript, immediately following the paragraph in which the figure is first cited (read order)****. Do not include captions as part of the figure files themselves or submit them in a separate document.”*

16. Figures: All figures should clearly refer to Groups 1, 2 or 3.

We specified Groups 1, 2 and 3 on updated figures and captions.

Discussion

17. Line 370: replace “8 to 9.5 min” to “7 to 10.5 min.” Read and cite Welsh et al (2002), *Adv Neurol*, Chapter 32 to see quantitative description of this.

As requested by the reviewer, we replaced this data in our manuscript and cited the reported reference.

18. Line 373-375. The intensity of the myoclonic jerk in the mechanical cardiac arrest model of PHM does not peak at day 4 post surgery. The best quantitative measures of the PHM audiogenic jerk by capacitive sensors and multi-muscle EMG indicate that it is most severe 2 days post anoxia/ischemia, and decreases nearly linearly over the following 3 weeks. Read and cite Welsh et al (2002), *Adv Neurol*, Chapter 31 to see high-res, quantitative analyses that were designed to be more sensitive than the myoclonus score.

As requested by the reviewer, we specified this data in our manuscript and cited the reported reference.

19. Lines 376-377. Rats having undergone mechanical cardiac arrest leading to PHM are often very ataxic for weeks following anoxia, perhaps due to action-induced myoclonic jerks. Although that was not well studied, those rats show slower and jerkier voluntary (i.e. conditioned) arm movements during the period that they show audiogenic myoclonus (see and cite Welsh et al (2002) *Adv Neurol*, Chapter 31). To a degree, those behavioral observations counter the authors’ suggestion here that the mechanical cardiac arrest model lacks face validity to human PHM because it induces audiogenic jerks only. This should be stated.

As requested by the reviewer, we specified this data in our manuscript and cited the reported reference.

20. Line 389. See Welsh et al (2002) *Adv Neurol* Chapter 32 and a growing clinical literature about cerebellar involvement in PHM as a complement to M1 involvement. EMG data suggests that rat cardiac arrest PHM is driven by a brainstem generator (see Welsh et al Adv Neurol Chapter 31).

As requested by the reviewer, we specified this possible involvement of subcortical structures in posthypoxic myoclonus.

21. Line 441. Replace “note” with “not.”

Typo corrected.

22. The Discussion is greatly improved from the previous version and appreciated.

We thank the reviewer for this comment.
