# Supplementary material for: Towards new animal models of pure hypoxic Lance-Adams syndrome: Negative results
Source: PLoS One. 2026 Jul 8;21(7):e0317638. doi: 10.1371/journal.pone.0317638 (PMC13345465; doi:10.1371/journal.pone.0317638)
Supplement: Attachment — Submitted filename: Response to Reviewers.docx [file pone.0317638.s003.docx]

**Reviewer #1: This is an interesting study. Although I am not close to the subject, I could follow the ms and I think that the rat model and the system that the authors are developing can be useful. I have no suggestions for changes.**

We thank the reviewer for this comment.

**Reviewer #2: The authors attempted to re-create a model to study LAS using various anoxia and hypoxia controlled procedures. However, the authors did not demonstrate the necessary phenotypic responses often observed in human patients nor were they able to replicate previously established results.**

**Comments/questions:**

**What current treatments are available?**

The current treatments for patients with Lance-Adams syndrome (LAS) are pharmacological anti-myoclonic treatments, mainly benzodiazepines and anti-seizure medications. As we reported in an up-to-date systematic review, the three most commonly used drugs in LAS patients were clonazepam, valproate, and levetiracetam (work under review in another journal). Moreover, in the cohort we recently published, we showed that levetiracetam, valproate, clonazepam, zonisamide, and perampanel showed better long-term retention rates (*Vellieux et al., Neurology, 2024, DOI: 10.1212/WNL.0000000000209994*).

Non-pharmacological approaches have also been reported in medication-refractory patients, including deep brain stimulation, especially in the globus pallidus internus, and iterative electroconvulsive therapy, as we previously reported in one of our patients (*Vellieux et al., Brain Stimulation, 2023, DOI: 10.1016/j.brs.2023.03.004*).

Regarding animal models of post-hypoxic myoclonus, myoclonic jerks in post-hypoxic rats decreased with valproate, clonazepam, 5-hydroxytryptophan (*Truong et al., Movement Disorders, 1994, DOI: 10.1002/mds.870090214*), piracetam, levetiracetam, brivaracetam (*Tai and Truong, Journal of Neural Transmission, 2007, DOI: 10.1007/s00702-007-0788-3*), and gabapentin (*Kanthasamy et al., European Journal of Pharmacology, 1996, DOI: 10.1016/0014-2999(95)00741-5*).

***We added this point in the Introduction of the revised manuscript***: “Several anti-myoclonic treatments are available for the patients, including pharmacological anti-seizure medications, such as clonazepam, valproate, and levetiracetam, and neuro-stimulation approaches in medication-refractory patients, such as deep brain stimulation or electroconvulsive therapy (2,4–6).”

**The weight and the sex of the animal can drastically affect oxygen tolerance and the subsequent downstream physiological and molecular responses. Is there a reason why only male rats were used?**

We chose male Sprague-Dawley rats weighing around 250-300 g because this type of animal was used in previous animal models of post-hypoxic myoclonus (*Truong et al., Movement Disorders, 1994, DOI: 10.1002/mds.870090214; Truong et al., Movement Disorders, 2000, DOI: 10.1002/mds.870150706*).

***We added this point in the Discussion of the revised manuscript***: “The two rodent models of LAS employed either pharmacological or mechanical ischemic procedures on male Sprague-Dawley rats weighing 250-300 g (10). […] We chose male Sprague-Dawley rats because they were previously employed for LAS models and CA rodent models (18). Young adult rats around 250-300 g were easy to handle, especially during intubation.”.

**Why wasn't the same anesthetic regiment used as hypoxia non-intubated animals? Extended exposure to isoflurane has significant effects on heart rate, which is an important parameter measured in your study. The authors must clearly demonstrate these differences and solidly prove that the reduced cardiac function is not due to extended isoflurane exposure.**

For the first group of hypoxic non-intubated animals, we could not use an inhalation sedation method with hypnotic gas because our homemade hypoxia chamber was not equipped with a closed gas circuit with a filtration system to collect the released gas. We thus chose to use an intraperitoneal injection with hypnotic drugs for sedation, which is a usual administration route in rodents, and also considering the excellent handling skills of our team. We used ketamine and xylazine, also aware of the possible xylazine-induced bradycardia. However, it represents a classical sedation protocol in rodents, already used in former animal models of post-hypoxic myoclonus (*Truong et al., Movement Disorders, 1994, DOI: 10.1002/mds.870090214; Truong et al., Movement Disorders, 2000, DOI: 10.1002/mds.870150706*).

For the second group of experiments in anoxic intubated animals, we took advantage of the animals’ ventilation circuit to use gas inhalation for sedation, especially isoflurane, which presents a moderate cardio-respiratory depression compared to xylazine. Gas anesthesia is usually safe because the sedation level can easily be controlled during maintenance, and anesthesia risks are usually very low. In most cardiorespiratory arrest animal models, anesthesia in small animals such as rodents is carried out using narcotic gases such as isoflurane (*Yu et al., Frontiers in Neuroscience, 2023, DOI: 10.3389/fnins.2022.1087725*). During our experiments, we first proceeded to sedation induction with 3% isoflurane in a dedicated induction chamber, then proceeded to sedation maintenance (after intubation) with 1.5% isoflurane. We then monitored the heart rate of our animals during the first few minutes before performing anoxia to ensure the absence of bradycardia. Moreover, in the control group, animals were also subjected to sedation with isoflurane for 10 minutes, and none of them showed bradycardia or unexpected death. Finally, some authors showed that the heart rate of Sprague-Dawley rats (12–16 weeks old and weighing 320±24 g) was relatively high and stable during sedation with isoflurane (induction 4% and maintenance 2% for 45 minutes) (*Murakami et al., Biological & Pharmaceutical Bulletin, 2014, DOI: 10.1248/bpb.b14-00012*).

***We added this point in the Discussion of the revised manuscript***: “We could not use an inhalation sedation with hypnotic gas because our homemade hypoxia chamber was not equipped with a closed gas circuit with a filtration system to collect the released gas. We therefore used an intraperitoneal injection with hypnotic drugs. […] In this second group of animals, we took advantage of the animals’ ventilation circuit to use gas inhalation for sedation, especially isoflurane, which presents a moderate cardio-respiratory depression compared to xylazine. Gas anesthesia is usually safe because the sedation level can easily be controlled during maintenance, and anesthesia risks are usually very low (21). In most cardiorespiratory arrest animal models, anesthesia in small animals such as rodents is carried out using narcotic gases such as isoflurane (18).”

**What parameters were used to determine the number of animals for each study? Please elaborate on the power analysis performed to make these determinations.**

First, it should be noted that there is no prevalence or incidence data regarding LAS in humans, especially the proportion of patients who suffered from chronic post-hypoxic myoclonus (i.e., LAS) after an anoxic/ischemic event. Moreover, in the previous experimental studies that developed an animal model of post-hypoxic myoclonus, the authors did not mention any precise data about the survival rate of the experiments and the proportion of animals that suffered from myoclonic jerks after global cerebral ischemia. Given this absence of both human epidemiological data and previous validated data from former animal models, it was currently not possible to perform a conventional sample size calculation based on statistical power analysis. There is indeed no available information regarding the expected prevalence, survival rate, or the probability of successfully inducing the target phenotype in animals. Therefore, this study was designed as an exploratory (pilot) investigation, whose primary objective was to assess the feasibility and reproducibility of the model.

To comply with the ethical principles of the 3Rs (Replacement, Reduction, and Refinement), we have chosen to limit the number of animals used in this initial phase and to adjust our experimental protocol based on the results obtained as the procedures progressed: (i) for non-intubated hypoxic rats: sedative dosage, oxygen concentration in the gas cylinder used, gas inflow rate into the chamber, and (ii) for intubated anoxic rats: continuous vs. intermittent anoxia, duration of anoxia.

Our objectives were thus to observe whether the pathological phenotype can be induced, to evaluate the onset, severity, and reproducibility of myoclonic jerks, to monitor survival and welfare-related endpoints, and to estimate the variability of biological responses for future power calculations.

In conclusion, this preliminary dataset was initially thought to allow for a rigorous statistical justification of sample size in subsequent confirmatory experiments to obtain meaningful exploratory data and minimize unnecessary animal use.

**There are molecular differences between hypoxia/anoxia and ischemia. Can the authors elaborate on what's already know in literature? It would be useful to measure these factors (ie. blood cytokines, some histological analysis on tissues), to establish a strong baseline on whether your model can recapitulate what happens during LAS in humans. These molecular changes are necessary to confirm whether the model (to be created) causes similar pathologies to humans.**

We agree with the reviewer that the biochemical and cellular mechanisms involved in neuronal damage caused by hypoxia/anoxia and ischemia may vary. This variability may lead to heterogeneous biochemical consequences in the micro-environment of neurons, especially in energy depletion, ion homeostasis, cell membrane integrity, oxidative stress, excitotoxicity, and cell death pathways (*Lipton, Physiological Reviews, 1999, DOI: 10.1152/physrev.1999.79.4.1431*). We can mention a few examples. Experimentally, hypoxia and ischemia produce different physiological responses in the brain. Ischemia is associated with a marked elevation in extracellular glutamate concentrations, and glutamate antagonists attenuate the neuropathologic injury. Hypoxia (PaO2 20 mmHg for 20 minutes), without ischemia, is neither associated with an elevation in extracellular glutamate concentrations nor with neuropathologic changes in rats, meaning that hypoxia without ischemia seems better tolerated than ischemia by the brain (*Pearigen et al., Brain Research, 1996, DOI: 10.1016/0006-8993(96)00215-6*). Moreover, gene expression is injury-specific as well. Although ischemia induces robust expression of the stress proteins HSP72 and HSP32, hypoxia alone induces neither gene product (*Pearigen et al., Brain Research, 1996, DOI: 10.1016/0006-8993(96)00215-6; Bergeron et al., Developmental Brain Research, 1998, DOI: 10.1016/s0165-3806(97)00169-7)*. The absence of pathologic sequelae of hypoxia without ischemia has been described in humans as well (*Gray and Horner, JAMA, 1970; Rie and Bernad, Neurology, 1980*). The relative benignity of hypoxia without ischemia was also demonstrated in lightly anesthetized primates in a 3.2% oxygen with nitrogen atmosphere. Neuropathologic changes were rare, and when seen, occurred in arterial border zones only (*de Courten-Myers et al., Stroke, 1985, DOI: 10.1161/01.str.16.6.1016).* Similar results occurred in hypoxic but normotensive cats maintained with a PO2 of 17 mmHg for 25 minutes. All animals were normal clinically and neuropathologically on recovery (*Auer and Mivamoto, Soc Neurosci Abstracts, 1995*). However, LAS is associated with prolonged and profound anoxic-ischemic events leading to clinical symptoms, mainly action myoclonus, and to histopathological abnormalities, although nonspecific (*Castaigne et al., Revue Neurologique, 1964; Masson et al., Revue Neurologique, 1975; De Léan et al., Advances in Neurology, 1986; Hauw et al., Advances in Neurology, 1986*).

In our previously mentioned work, which is under review in another journal, we showed that two-thirds of patients with LAS reported in the literature suffered from a primary hypoxic respiratory event, leading or not to cardiac arrest and thus global cerebral ischemia. For this main reason, we tried to induce a novel animal model of chronic post-hypoxic myoclonus using pure hypoxia/anoxia, leading to or not to bradycardia and cardiac arrest in our animals.

***We added this point in the Discussion of the revised manuscript***: “Patients with LAS indeed mainly suffered from a primary hypoxic respiratory event (2). The biochemical and cellular mechanisms involved in neuronal damage caused by hypoxia/anoxia and ischemia may vary (13). This variability may lead to heterogeneous biochemical consequences in the micro-environment of neurons, for example, on extracellular glutamate concentrations (14), gene expression (14,15), or neuropathological changes (16,17).”

**Figure need to be uploaded with higher quality. Figure 5 was not legible at all.**

The figures embedded in the PDF manuscript are indeed of low quality. However, a high-resolution version of each figure is available via the download link located at the top-right corner of each figure.
