# Supplementary material for: Towards new animal models of pure hypoxic Lance-Adams syndrome: Negative results
Source: PLoS One. 2026 Jul 8;21(7):e0317638. doi: 10.1371/journal.pone.0317638 (PMC13345465; doi:10.1371/journal.pone.0317638)
Supplement: Attachment — Submitted filename: Response to Reviewers PLOS ONEvn.docx [file pone.0317638.s004.docx]

**You will find in this document our responses (in blue) to the Reviewer #3’s comments (in black).**

The manuscript is well-written and describes important exploratory attempts.

The authors aimed to establish a reproducible animal model of Lance-Adams syndrome (LAS), but all hypoxic or anaerobic treatment regimens were unsuccessful in inducing a myoclonic phenotype. Thus, this study failed to establish a valid disease model, which limits its translational medical value.

We would like to emphasize that the primary aim of our article was to share with the scientific community our **« negative results »,** specifically to prevent other research groups from repeating the two experimental protocols we tested to establish a model of post-anoxic myoclonus, or Lance-Adams syndrome. Our manuscript was explicitly submitted under the category “Research Article – Negative Results.”

To clearly emphasize the objective of our report, we have now modified the title of our manuscript as: “Towards new animal models of pure hypoxic Lance-Adams syndrome: **negative results**”.

Publishing such data helps reduce unnecessary duplication of animal experiments, enhances transparency, and ultimately advances the search for more effective and ethically sound models of this devastating condition.

We are continuing to develop a model of Lance–Adams syndrome using more invasive strategies. It is important to share with the scientific community that the two protocols we rigorously applied were not sufficient to elicit post-anoxic myoclonus.

The high mortality rate of 26% to 50% among the protocols is not only ethically controversial, but also undermines the feasibility of the proposed model. This is contrary to the stated goal of developing “less invasive methods with lower mortality”.

We fully agree that both procedures failed to induce a reproducible phenotype and were associated with elevated mortality. However, contrary to Reviewer 3’s concerns, these protocols were not ethically controversial. Both were reviewed and approved by the local Ethics Committee Charles Darwin CEEACD/N°5 and by the French Ministry of Research (APAFIS approvals #35162-2022060716206563 and #43666-2023083015044880). Given the severity of human anoxic injuries and the urgent need for accurate preclinical models, the potential risks of these procedures were justified and carefully managed. Importantly, high mortality rates are unfortunately consistent with the clinical reality of severe anoxia in patients.

Data on the mortality has been removed from the Abstract.

The use of only male Sprague-Dawley rats, although based on precedent, ignores sex differences in oxygen tolerance and neurophysiology.

We agree with the reviewer that our choice to perform our experiments only in male Sprague-Dawley rats is debatable and that it may influence oxygen tolerance and subsequent physiopathological responses. However, our experiments were fully exploratory, only based on previous successful ischemic procedures of other teams (Truong et al., Movement Disorders, 1994, DOI: 10.1002/mds.870090214; Truong et al., Movement Disorders, 2000, DOI: 10.1002/mds.870150706). We took advantage of these successful procedures to reuse the same rat strain, age and sex. If we had succeeded in inducing the desired myoclonic phenotype in male rats, we would obviously have replicated our experiments on females.

This point has been added in the Discussion.

Sample size calculations were not performed, and the basis for an exploratory “pilot study” was weak in view of the high mortality rate and lack of phenotypes.

First, it should be noted that there is no prevalence or incidence data regarding LAS in humans, especially the proportion of patients who suffered from chronic post-hypoxic myoclonus (i.e., LAS) after an anoxic/ischemic event. Moreover, in the previous experimental studies that aimed to develop an animal model of post-hypoxic myoclonus, the authors did not mention any precise data about the survival rate of the experiments and the proportion of animals that suffered from myoclonic jerks after global cerebral ischemia. Given this absence of both human epidemiological data and previous validated data from former animal models, it was currently not possible to perform a conventional sample size calculation based on statistical power analysis. There is indeed no available information regarding the expected prevalence, survival rate, or the probability of successfully inducing the target phenotype in animals. Therefore, this study was designed as an exploratory (pilot) investigation, whose primary objective was to assess the feasibility and reproducibility of the model.

To comply with the ethical principles of the 3Rs (Replacement, Reduction, and Refinement), we have chosen to limit the number of animals used in this initial phase and to adjust our experimental protocol based on the results obtained as the procedures progressed: (i) for non-intubated hypoxic rats: sedative dosage, oxygen concentration in the gas cylinder used, gas inflow rate into the chamber, and (ii) for intubated anoxic rats: continuous vs. intermittent anoxia, duration of anoxia.

Our objectives were to observe whether the pathological phenotype can be induced, to evaluate the onset, severity, and reproducibility of myoclonic jerks, to monitor survival and welfare-related endpoints, and to estimate the variability of biological responses for future power calculations.

In conclusion, this preliminary dataset was initially thought to allow for a rigorous statistical justification of sample size in subsequent confirmatory experiments to obtain meaningful exploratory data and minimize unnecessary animal use.

Different anesthetic regimens were used in different groups, with confounding effects on cardiovascular responses. The rationale for terminating hypoxia at a specific heart rate threshold is not well documented.

For the first group of hypoxic non-intubated animals, we could not use an inhalation sedation method with hypnotic gas because our homemade hypoxia chamber was not equipped with a closed gas circuit with a filtration system to collect the released gas. We thus chose to use an intraperitoneal injection with hypnotic drugs for sedation, which is a usual administration route in rodents, and also considering the excellent handling skills of our team. We used ketamine and xylazine, also aware of the possible xylazine-induced bradycardia. However, it represents a classical sedation protocol in rodents, already used in former animal models of post-hypoxic myoclonus (*Truong et al., Movement Disorders, 1994, DOI: 10.1002/mds.870090214; Truong et al., Movement Disorders, 2000, DOI: 10.1002/mds.870150706*).

For the second group of experiments in anoxic intubated animals, we took advantage of the animals’ ventilation circuit to use gas inhalation for sedation, especially isoflurane, which presents a moderate cardio-respiratory depression compared to xylazine. Gas anesthesia is usually safe because the sedation level can easily be controlled during maintenance, and anesthesia risks are usually very low. In most cardiorespiratory arrest animal models, anesthesia in small animals such as rodents is carried out using narcotic gases such as isoflurane (*Yu et al., Frontiers in Neuroscience, 2023, DOI: 10.3389/fnins.2022.1087725*). During our experiments, we first proceeded to sedation induction with 3% isoflurane in a dedicated induction chamber, then proceeded to sedation maintenance (after intubation) with 1.5% isoflurane. We then monitored the heart rate of our animals during the first few minutes before performing anoxia to ensure the absence of bradycardia. Moreover, in the control group, animals were also subjected to sedation with isoflurane for 10 minutes, and none of them showed bradycardia or unexpected death. Finally, some authors showed that the heart rate of Sprague-Dawley rats (12–16 weeks old and weighing 320±24 g) was relatively high and stable during sedation with isoflurane (induction 4% and maintenance 2% for 45 minutes) (*Murakami et al., Biological & Pharmaceutical Bulletin, 2014, DOI: 10.1248/bpb.b14-00012*).
