# Supplementary material for: Towards new animal models of pure hypoxic Lance-Adams syndrome: Negative results
Source: PLoS One. 2026 Jul 8;21(7):e0317638. doi: 10.1371/journal.pone.0317638 (PMC13345465; doi:10.1371/journal.pone.0317638)
Supplement: Attachment — Submitted filename: Response_to_Reviewers_auresp_3.docx [file pone.0317638.s006.docx]

**You will find in this document our responses (in blue) to the Reviewers’ comments (in black).**

**Reviewer #4:** This manuscript reports the behavioral effects of three approaches for inducing hypoxic injury in rats. The primary outcome is a myoclonus score, depicted in Figure 5. No effect of the hypoxic insults was detected. This reporting of a neutral result is always difficult, because of the high burden of proving that failure to detect something means that it is not present. It is clear that the insult was sufficiently severe, because it conveyed high mortality.
There is a need for an animal model of post-hypoxic myoclonus. Aside from the cited papers from the 1990's, there has not been any work with such a model. One aspect of myoclonus that this paper glosses over is that status myoclonus observed in the early time after hypoxia may be unrecoverable, but be part of the clinical spectrum that includes LAS (e.g. https://pubmed.ncbi.nlm.nih.gov/24286857/ and <https://pubmed.ncbi.nlm.nih.gov/27351833/>)

We agree with the reviewer that our medical and scientific community does need an animal model for post-hypoxic myoclonus. This extremely disabling condition represents a burden for surviving patients and their relatives in a context of no efficient therapy. An animal model would be of great help to better understand the pathophysiology of this neurological disorder facing many unmet medical needs.

We also agree with the reviewer that Lance-Adams syndrome belongs to the broader category of post-hypoxic myoclonic syndromes and that it represents its ‘chronic’ form associated with a good prognosis in terms of mortality, contrary to the ‘acute’ form of post-hypoxic myoclonus which is associated with a very poor prognosis. In our recent review in *Brain Communications* (DOI: 10.1093/braincomms/fcaf329), we highlighted this continuum outlining that few patients who will further develop LAS may previously show myoclonus during coma, with a different semiology. The diagnosis of LAS is therefore challenging in comatose patients. Some authors define as ‘acute’ post-hypoxic myoclonus any patient with myoclonus’ onset during the first hours or days after the anoxic event. The prognosis is heterogeneous among these patients. However, some other patients with ‘acute’ post-hypoxic myoclonus may then develop Lance-Adams syndrome, for some defined as ‘chronic’ post-hypoxic myoclonus after regaining consciousness, portending a far better prognosis, even if they presented with generalized myoclonus in the comatose post-anoxic period.

Our goal was to develop an animal model of the chronic form of post-hypoxic myoclonus, i.e. Lance-Adams syndrome, occurring in survivors of anoxic brain insult. This animal model would be a great help to confirm (or rule out) our pathophysiologic hypothesis obtained from the study of our human cohort, recently published in *Neurology* (DOI: 10.1212/WNL.0000000000209994).

1. The statistical analysis is vague. A few tests are named, but the actual comparisons are poorly defined. The key outcome is Figure 5: a time series before and after an intervention of repeated measurements in individual animals. One simple analysis would be to model the score, Y~ A(time)+B(group)+C(group x time)+error, accounting for clustering within the animal. To simplify, time could even be pre/post intervention. In that sort of model, the primary comparison would be whether coefficients for group or group x time differ from zero. Another approach would be to collapse all the observations before and after the injury (ignoring sequential time), and compare scores pre/post, again accounting for repeated measures.

2. If the result is neutral, then the results should be expressed in terms of what difference between groups was observed with a confidence interval on that difference. It is not sufficient to say that a p-value was < or > some threshold, since p-values are not measures of effect size and are influenced by number of observations and choice of test. For example, the results might read: "The mean daily score for sham animals was 10±3 and for anoxic animals was 15±4 with a mean difference of 5 (95% CI -4 to 10)." This form will allow future researchers to know how large of a difference has been reliably excluded by these data.

We appreciate the reviewer’s suggestion to employ a linear mixed-effects model (LMM) to account for the repeated-measures structure of our data. Below, we detail the implementation, results, and robustness checks of this approach.

We fitted the following LMM to our data: score ~ time * group + (1 | rat_id)

- “score” = myoclonus score (primary outcome).
- “time” = days before and after hypoxic or anoxic protocol
- “group” = experimental condition controls vs experimental protocol (hypoxia, continuous anoxia or intermittent anoxia).
- “(1 | rat_id)” = random intercept for each animal to account for within-subject correlation.

Analyses were conducted in R (v 4.5.1) using the “lme4” (v 1.1.37), “lmerTest” (v3.1.3), and “emmeans” (v2.0.0) packages. Model convergence was confirmed via diagnostic plots (residuals vs. fitted values, Q-Q plots) and the absence of singularity warnings.

The model output is summarized in the Tables below.

1/ Hypoxia vs controls (Table 1)

| **Effect** | **Estimate (SE)** | **95% CI** | **df** | **p-value** | **Sig.** |
| --- | --- | --- | --- | --- | --- |
| Time | -1.8833 (0.3188) | [-2.5118, -1.2547] | 1 | <0.001 | *** |
| Group | 0.2689 (5.3754) | [-11.1293, 11.6672] | 1 | 0.9607 | ns |
| Time:group | 0.523 (0.3659) | [-0.1982, 1.2443] | 1 | 0.1543 | ns |

For the comparison of hypoxia versus controls, we found a main effect of time (p<0.001), suggesting a significant modification of the myoclonus score over time. The statistical model did not show significant global difference between the hypoxic group and the control group. We also did not demonstrate any significant interaction between the time and the group.

2/ Continuous anoxia vs controls (Table 2)

| **Effect** | **Estimate (SE)** | **95% CI** | **df** | **p-value** | **Sig.** |
| --- | --- | --- | --- | --- | --- |
| Time | -0.1795 (0.065) | [-0.3073, -0.0517] | 1 | <0.001 | *** |
| Group | 0.0729 (1.4842) | [-2.9862, 3.132] | 1 | 0.9612 | ns |
| Time:group | -0.1323 (0.0772) | [-0.2841, 0.0195] | 1 | 0.0874 | ns |

For the comparison of continuous anoxia versus controls, we found a main effect of time (p<0.001), suggesting a significant modification of the myoclonus score over time. The statistical model did not show significant global difference between the continuous anoxia group and the control group. We also did not demonstrate any significant interaction between the time and the group.

3/ Intermittent anoxia vs controls (Table 3)

| **Effect** | **Estimate (SE)** | **95% CI** | **df** | **p-value** | **Sig.** |
| --- | --- | --- | --- | --- | --- |
| Time | -0.0789 (0.0536) | [-0.1843, 0.0265] | 1 | <0.001 | *** |
| Group | 1.7031 (2.1795) | [-2.8483, 6.2546] | 1 | 0.4439 | ns |
| Time:group | -0.098 (0.0656) | [-0.227, 0.031] | 1 | 0.1360 | ns |

For the comparison of intermittent anoxia versus controls, we found a main effect of time (p<0.001), suggesting a significant modification of the myoclonus score over time. The statistical model did not show significant global difference between the intermittent anoxia group and the control group. We also did not demonstrate any significant interaction between the time and the group.

We added all these data to the updated version of the manuscript, in the Material & Methods section as in the Results section.

3. Figure 5. The values graphed are mean (SD), but this ordinal scale is bounded by zero (values cannot be negative). It would be more appropriate to graph median (IQR) for this type of score. This figure would be more clear if the y-axis was moved to the left: a dashed line could still separate pre/post or indicate the intervention day.

As requested by the reviewer, we changed the values on the three plots of Figure 5. In the new version of the manuscript, you will find the median (error bar: interquartile) on a plot with y-axis moved to the left and a line at day 0 separating pre- and post-protocol.

4. There are a few odd phrasings: line 136 "brutal 100% N2 ventilation" - the word "brutal" implies violence, and I think the authors might have meant "severe". Truly, no adjective is required since "100% N2 ventilation" speaks for itself. lines 276-7 "we observed that rats almost systematically
died" - "systematically" probably should be "universally" or "always". If this can be a number (90% died) it would be more informative.

We agree with the reviewer and have deleted the words ‘brutal’ line 136 and ‘brutally’ line 133.

We agree with the reviewer and have specified that “we observed that 75% of rats died when the HR was < 140-150 beats/min…”.

5. The discussion should mention two possibilities that may prevent the success of this effort. First, post-hypoxic myoclonus might be a sequella of severe brain injury which would require post-hypoxia intensive care. These models with spontaneously-breathing rats may be unable to detect the myoclonus, whereas more heroic or prolonged ventilations of some of the animals who died in these models might have revealed myoclonus. Second, the rat has an agyric cortex with substantial differences from humans. It is possible that the myoclonus syndrome is a feature of primate or more gyrencephalic brains.

We thank the reviewer for these two important comments.

1/ The first hypothesis of the reviewer is that a more prolonged resuscitation of animals would bring more survivors and then more animals prone to developing post-hypoxic myoclonus. We agree with this comment. We had discussed at the end of our article some methods that would refine our manipulations: capnograph for capnography-guided procedures, intravascular arterial and venous catheters for invasive monitoring of arterial blood pressure and administration of catecholamines.

Moreover, we agree that post-anoxic resuscitation itself, including reoxygenation and mechanical ventilation parameters, may contribute to secondary brain injury and should therefore be considered in the pathophysiological interpretation of post-anoxic phenomena. Experimental and clinical studies have shown that hyperoxia following return of spontaneous circulation can exacerbate oxidative stress and neuroinflammation. In animal models of cardiac arrest, ventilation with 100% oxygen after resuscitation has been associated with worse neurological outcomes compared with normoxic strategies (Liu et al., 1998, *Stroke*). More recent experimental work has also demonstrated increased inflammatory and apoptotic signaling in the brain following hyperoxic resuscitation (Aoki et al., 2023, *Scientific Reports*). In parallel, clinical data suggest that extreme PaCO₂ values, particularly severe hypocapnia, are associated with poorer neurological outcomes after cardiac arrest (Okada et al., 2022, *Journal of Clinical Medicine*; Xue et al., 2025, *Anaesthesia Critical Care & Pain Medicine*). These findings support the concept that post-resuscitation management may modulate the extent of global cerebral injury through oxidative and cerebrovascular mechanisms.

Importantly, the current literature does not identify reoxygenation toxicity per se as a specific mechanistic trigger of post-hypoxic myoclonus. While oxidative stress and inflammation may contribute to the overall severity of neuronal damage, available evidence does not support a direct and selective role of hyperoxia or ventilator-induced effects in generating myoclonic activity. Rather, these factors appear to modulate injury severity at a global level.

2/ The smooth lissencephalic cortex of the rat differs markedly from the folded gyrencephalic cortex of humans and primates, which may impact the capacity of the rodent brain to exhibit complex network phenomena such as sustained involuntary movements after global hypoxic injury. These gyrification differences correlate with quantitative and qualitative distinctions in cortex organization. Non-human primates and humans exhibit a larger proportion of isocortex and expanded prefrontal regions than rodents, which are associated with higher-order motor control, complex sensory integration, and sustained cognitive processing not paralleled in rodent models (Zhang et al., 2023, *National Science Review*). Post-hypoxic myoclonus in humans, as a gyrencephalic species, may likely engage distributed cortical loops involving extensive sensorimotor territories, including motor, premotor and supplementary motor areas that are disproportionately expanded in gyrencephalic cortices (Vellieux et al. *Neurology*, 2024). The absence of sulci and extensive gyri in rats might limit the emergence or expression of certain network-level disturbances that underlie sustained myoclonic syndromes after diffuse hypoxic injury in humans. Therefore, while basic neuronal and synaptic processes are conserved across mammals, the topological and network-level differences associated with gyrification and cortical expansion should be acknowledged as limitations in modelling certain human clinical phenotypes using rodents.

However, some authors have reported results of myoclonic phenotypes in rodents through various contexts, for example as already specified post-hypoxic myoclonus (Truong et al., 2000*, Movement Disorders*), but also progressive myoclonic epilepsy (Buzzi et al., 2012, *Neurobiology of Disease*) or juvenile myoclonic epilepsy (Velisek et al., 2011, PLoS One; Arain et al., 2016, *Neurobiology of Disease*), outlining the fact that it seems realistic to try to create a myoclonic phenotype in rodents.

We added these two points of discussion to the dedicated section of the new version of the manuscript.

**Reviewer #5:** PLOS One – Towards new animal models of pure hypoxic Lance-Adams syndrome: Negative Results

As was pointed out in previous reviews, this manuscript describes three logical, but ineffective, strategies to induce post-hypoxic startle myoclonus in rats, either by ambient replacement of oxygen with nitrogen in an enclosed chamber or by two different intubated ventilation regimes with 100% nitrogen. The previous review pointed out that the translational value of the manuscript is limited by negative outcomes (i.e., no rat phenotype resulted). Technical and statistical considerations also were raised. Yet, I am not convinced that those arguments are relevant by themselves for deciding to reject this manuscript.
If PLOS is amenable to publishing negative outcomes, I would ask them to consider the manuscript more fully because there is significant value, which I explain below. In addition, I believe that the manuscript can be improved by the authors to further increase value to those interested in this field, if the authors would more deeply discuss the clinical and scientific implications of their negative findings.

1. I have performed dozens of cardiac arrest procedures in rats using the mechanical metal-hook procedure that the authors consider the gold-standard to produce post-hypoxic startle myoclonus (disseminated by Truong et al., which the authors cite). That procedure is tricky and requires significant experience of a skilled investigator to reliably produce startle myoclonus with minimum mortality. Also, as an investigator learns the procedure, there can be significant ramp-up mortality. Not noted in the manuscript, the procedure can induce neurological symptoms adding to startle myoclonus (ataxia, acute seizures, paraplegia in cases), for which some local institutional veterinarians, Animal Care and Use Committees, and administrators are predisposed to view skeptically, even though humans suffer immensely with a much worse condition. It is an obvious and a worthy goal to identify a rat procedure that might be less invasive, less impairing, and produces less mortality, but still produces a clinically-relevant myoclonus phenotype using the methods described in this manuscript.
To try to achieve that goal, we, and perhaps others, tested many closely identical procedures in this manuscript years ago, with the identical negative outcomes described. It would be very useful if anoxia sans acute cardiac arrest effectively produced startle myoclonus in rats. However, as systematically evidenced in this manuscript, and by my and others’ prior unpublished efforts, this is not true.
Thus, given that future investigators may travel down this path and repeat, yet again, the negative experiments described here, I recommend publishing this manuscript. This is especially so given one Reviewer’s dissatisfaction that there was a degree of mortality using the approaches described. I’m less concerned by that rat mortality (there is no indication that those rats were treated inhumanely; the experiments were performed according to guidelines and approvals), given that human morbidity and mortality is far greater and solutions to this magnitude of brain hypoxia in the clinic are in fact needed. Publishing this manuscript would help ensure future investigators to direct their effort and resources elsewhere, benefitting everyone.

We sincerely thank the reviewer for these valuable insights and constructive feedback regarding our manuscript. We appreciate his/her extensive experience with cardiac arrest models and your thoughtful comments.

We would like to emphasize that the reviewer has perfectly understood our research question. Our medical and scientific community do need an animal model for post-hypoxic myoclonus. This extremely disabling condition represents a burden for surviving patients and their relatives in a context of no efficient therapy. An animal model would be of great help to better understand the pathophysiology of this neurological disorder facing many unmet medical needs.

We acknowledge that our negative results may be disappointing, but we agree with the Reviewer’s assessment that they provide important information for the scientific community. The comments on technical challenges and mortality associated with the mechanical hook procedure are well-taken and highlight why alternative approaches are worth exploring despite our unsuccessful outcomes.

2. I was unconvinced by a previous Reviewer’s point that the value of the study was lessened by the lack of power analysis. Obviously, this is a pilot and no estimates of variance could have informed the experiments up front. Performing the procedures on a defined number of rats without sign of obvious myoclonic effect is likely sufficient to justify concluding that the procedures do not produce startle myoclonus of a magnitude approaching following anoxic cardiac arrest, without having to further establish that a larger group size would be needed to statistically validate a possible effect that is not obvious visually. Startle myoclonus in rats is very visually obvious.

We thank the reviewer for this comment. Indeed, we could not calculate a precise number of animals to use for our protocols, because of previous similar experiments lacking. Our pilot protocols were purely exploration and, among others, aimed to determine the parameters of hypoxia/anoxia leading to a myoclonic phenotype.

3. It is, in fact, valuable to know that mortality using this non-invasive anoxia procedure was high, while the incidence of startle myoclonus was zero or near zero. The former illustrates the procedures were sufficient to evoke severe pathophysiology, but not of the type that produces startle myoclonus. This further justifies publishing the manuscript, in my view. This point could be expounded upon further by the authors.

We agree with the reviewer. We must find the right experimental balance between, on the one hand, inducing anoxia severe enough to cause neuronal damage leading to a myoclonic phenotype, and on the other hand, avoiding anoxia that is too severe, as it could result in high mortality or excessively severe sequelae that would prevent proper evaluation of the animals’ phenotype. Several other experimental parameters must therefore be considered in future experiments, such as changes in carbon dioxide levels, effects of post-anoxia ventilation.

The publication of our results would help inform the discussion on the most appropriate way to generate a viable post-anoxic animal model with chronic myoclonus and a phenotype that more closely resembles that of humans and their action myoclonus (rather than reflex myoclonus).

These points have been added to the Discussion of the revised manuscript.

4. I believe that the manuscript would be improved and that its value would be increased immensely if the authors would rewrite their Discussion to address what they may believe are the differences in pathophysiology between anoxia alone vs. anoxic/ischemic cardiac arrest in inducing the behavioral phenotype. As it stands now, the Discussion just recapitulates the procedures and negative outcomes, which are covered earlier and do not add value. The authors have thought long and hard about LA syndrome as evidenced in their prior review paper. I’d like the authors to address what they believe is the difference in pathophysiology between nearly fatal anoxia vs. ischemic/anoxic cardiac arrest, only the latter of which produces the startle myoclonus phenotype in rats.

We thank the reviewer for this highly valuable comment. We have substantially expanded the Discussion to delineate the key pathophysiological differences between pure anoxia (with maintained cerebral perfusion) and combined hypoxic/ischemic insults (as occurs in cardiac arrest) and how these may explain our findings. We may propose that the critical difference lies in the distinct effects of anoxia alone versus combined ischemia–anoxia on cortical microcircuit integrity, particularly the balance between pyramidal neurons and inhibitory interneurons within motor cortex networks. Moreover, we agree that the key issue raised by our negative results is not procedural, but mechanistic: ischemic–anoxic cardiac arrest produces startle myoclonus phenotype in rats, whereas nearly fatal pure anoxia as we designed it does not.

Global ischemia induces simultaneous deprivation of oxygen and glucose, rapid ATP collapse, extracellular glutamate accumulation, and prolonged depolarization (Pearigen*, Brain Research*, 1996). Brief ischemic episodes can cause a persistent depression of GABAergic interneuron function while pyramidal neurons exhibit relative hyperexcitability during reperfusion. In particular, parvalbumin-positive interneurons appear especially vulnerable to oxygen–glucose deprivation, with impaired recovery of inhibitory transmission (Povysheva et al., Neuroscience, 2019). This selective dysfunction of inhibitory interneurons creates a state of cortical disinhibition, favoring pathological hypersynchrony. In contrast, during pure anoxia with preserved perfusion, glucose delivery and metabolite clearance continue. Although neuronal firing rapidly decreases under severe hypoxia, the metabolic collapse is less abrupt and pure anoxia may produce a more global depression of cortical activity rather than a selective failure of inhibition. We may therefore hypothesize that ischemia preferentially disrupts inhibitory control within motor cortex microcircuits, whereas pure anoxia produces more homogeneous network suppression. Moreover, post-hypoxic myoclonus in humans (Lance–Adams syndrome) is widely considered to have a cortical origin, with electrophysiological evidence implicating hyperexcitable sensorimotor cortex (Vellieux et al. *Neurology*, 2025; Vellieux et al., *Brain Communications*, 2025). We may thus propose that ischemic cardiac arrest creates the precise network conditions required for chronic motor cortex hyperexcitability: selective interneuron dysfunction leading to reduced GABAergic inhibition, pyramidal neuron survival in partially injured cortex leading to preserved output pathways, reperfusion-associated glutamatergic surges leading to excitotoxic priming, and subsequent maladaptive plasticity within corticospinal and cortico-subcortical loops. This combination could lead to a chronically unstable motor cortex, hypersensitive to sensory input and capable of generating stimulus-sensitive myoclonus. By contrast, in pure anoxia, the inhibitory tone may be relatively preserved and excitotoxic cascades may be less intense. Thus, the cortical microcircuit may never enter a disinhibited, hyperexcitable state sufficient to produce startle myoclonus. From a circuit-level point of view, ischemic arrest may also more profoundly affect cortico-thalamo-cortical and cortico-cerebellar loops through endothelial dysfunction, blood–brain barrier disruption, and reperfusion-induced inflammation. These secondary processes could destabilize oscillatory motor networks. Pure anoxia, in contrast, lacks the reperfusion phase and its associated oxidative and inflammatory cascades, potentially limiting long-term network reorganization.

In summary, we may hypothesize that ischemia produces selective inhibitory failure and maladaptive motor cortical plasticity, whereas pure anoxia induces a more global and potentially reversible depression of cortical function. This distinction may explain why only ischemic cardiac arrest induces the startle myoclonus phenotype in rodents.

Of note, we published two lengthy papers showing ischemic/anoxic cardiac arrest requires a threshold range of cardiac arrest parameters that produce rat PHM phenotype and that the behavior has a brainstem generator (Welsh et al Myoclonus and Paroxysmal Dyskinesias, Advances in Neurology 89, p. 307 and 331), uncited information that may be valuable to the authors. In our lab, PHM virtually never occurred when blood pressure without heart beat was not immediately lowered and sustained to below 10 mmHg for at least 7-7.5 min during complete anoxia, but not more than 9-9.5 min after which mortality was near 100%. Our own experiments indicated the neuron degeneration process occurred over 48-72 hr post-recovery, due to enhanced synaptic activity driven possibly by an upregulation in ionic channel conductances in rhythm-generating neurons. For those reasons, it became clear to us that even gradual reductions in brain oxygen, perhaps even sustained at a level near a very low threshold, would not produce PHM in rats – as reported here. It would be good for the authors to provide quantitative comments on this issue on why that is true.

Our experimental procedures highlight the importance of invasively monitoring arterial blood pressure through arterial catheterization. This would allow us to assess the dynamics of blood pressure changes in our model and to examine the correlation between the kinetics of blood pressure decline, mortality, and the phenotype of surviving animals. Furthermore, intravenous catheterization would enable the administration of vasopressor drugs.

These points have been included in the manuscript.

5. Finally, it would be helpful for the authors, given their clinical experience, to comment upon whether these rat efforts adequately model human post-hypoxic myoclonus. Does the exaggerated startle response have sufficient construct validity for human post-hypoxic myoclonus (in spite of absence of negative myoclonus and asterixis)? More importantly, based on the authors’ expertise, does the requirement of the rat’s startle myoclonus behavior for ischemic/anoxia lower (or increase?) its face validity to human post-hypoxic myoclonus? Is the rat perhaps not the best species for modeling human post-hypoxic myoclonus given these attributes, while retaining its value for basic science? If the authors would critically address those issues in the Discussion (rather than recapitulating methods), the value of this paper – despite the negative outcomes – would be greatly enhanced.

We were fully aware of the existing animal models of post-anoxic myoclonus, as reported in the introduction of our manuscript. However, we identified several limitations in these models, in particular:

1. The use of ischemia induced by cardiocirculatory arrest rather than pure hypoxia/anoxia, which limits its construct validity, given that in humans, two thirds of patients with Lance-Adams syndrome experienced an initial anoxic event: respiratory arrest/failure without cardiac arrest in 24% of patients and respiratory arrest/failure followed by cardiac arrest in 41% of patients (Vellieux G, Brain Communications, 2025);
2. The occurrence in animals of sound-induced muscle jerks rather than movement- or action-induced jerks, which limits its phenotypic and predictive validity, considering that in humans, action myoclonus is present in all patients, whereas reflex myoclonus is observed in 81% of patients (Vellieux G, Brain Communications, 2025).

Moreover, we chose the rat as the experimental species because, as indicated in our manuscript, the myoclonus rating scale was developed in rats, the rat’s hemodynamic parameters are relatively similar to those of humans, and previously developed animal models of post-anoxic myoclonus were established in rats, which are also an easy species to handle.

Finally, several authors have reported myoclonic phenotypes in rodents in various contexts, including post-hypoxic myoclonus (Truong et al., 2000, Movement Disorders), progressive myoclonic epilepsy (Buzzi et al., 2012, Neurobiology of Disease), and juvenile myoclonic epilepsy (Velíšek et al., 2011, PLOS ONE; Arain et al., 2016, Neurobiology of Disease), highlighting that it appears realistic to attempt to generate a myoclonic phenotype in rodents.

We specified more precisely these points in our manuscript.

Minor: Abstract: Rewrite the abstract for clarity to draw parallels between the 2 “groups” and 3 “procedures.” For instance, line 22 describes 2 experimental groups: hypoxia by cage replacement and hypoxia by intubation. Later (line 34) it describes 3 procedures: hypoxia without intubation; continuous anoxia by intubation; intermittent anoxia by intubation. This is confusing. I would describe the experiment as having 3 experimental groups, each with its own experimental procedure.

We agree with the reviewer and change the labels of our groups.
