# Supplementary material for: Towards new animal models of pure hypoxic Lance-Adams syndrome: Negative results
Source: PLoS One. 2026 Jul 8;21(7):e0317638. doi: 10.1371/journal.pone.0317638 (PMC13345465; doi:10.1371/journal.pone.0317638)
Supplement: Peer review history

# Peer review: Decision Letter 0

**DOI:** 10.1371/journal.pone.0317638.r001

**Authors:** Marcelo Hermes-Lima

28 Apr 2025

PONE-D-25-00224

Towards new animal models of pure hypoxic Lance-Adams syndrome

PLOS ONE

Dear Dr. Navarro,

Thank you for submitting your manuscript to PLOS ONE. After careful consideration, we feel that it has merit but does not fully meet PLOS ONE’s publication criteria as it currently stands. Therefore, we invite you to submit a revised version of the manuscript that addresses the points raised during the review process.

One of the referees made very strong arguments regarding the validity of your model. Please, answer carefully all questions presented by the referee so we can evaluate your work for a 2nd time.

Please submit your revised manuscript within Jun 12 2025 11:59PM. If you will need more time than this to complete your revisions, please reply to this message or contact the journal office at plosone@plos.org. When you're ready to submit your revision, log on to https://www.editorialmanager.com/pone/ and select the 'Submissions Needing Revision' folder to locate your manuscript file.

Please include the following items when submitting your revised manuscript:

- A rebuttal letter that responds to each point raised by the academic editor and reviewer(s). You should upload this letter as a separate file labeled 'Response to Reviewers'.
- A marked-up copy of your manuscript that highlights changes made to the original version. You should upload this as a separate file labeled 'Revised Manuscript with Track Changes'.
- An unmarked version of your revised paper without tracked changes. You should upload this as a separate file labeled 'Manuscript'.

If you would like to make changes to your financial disclosure, please include your updated statement in your cover letter. Guidelines for resubmitting your figure files are available below the reviewer comments at the end of this letter.

If applicable, we recommend that you deposit your laboratory protocols in protocols.io to enhance the reproducibility of your results. Protocols.io assigns your protocol its own identifier (DOI) so that it can be cited independently in the future. For instructions see: https://journals.plos.org/plosone/s/submission-guidelines#loc-laboratory-protocols. Additionally, PLOS ONE offers an option for publishing peer-reviewed Lab Protocol articles, which describe protocols hosted on protocols.io. Read more information on sharing protocols at https://plos.org/protocols?utm_medium=editorial-email&utm_source=authorletters&utm_campaign=protocols.

We look forward to receiving your revised manuscript.

Kind regards,

Marcelo Hermes-Lima, PhD

Academic Editor

PLOS ONE

Journal Requirements:

When submitting your revision, we need you to address these additional requirements.

1. Please ensure that your manuscript meets PLOS ONE's style requirements, including those for file naming. The PLOS ONE style templates can be found at https://journals.plos.org/plosone/s/file?id=wjVg/PLOSOne_formatting_sample_main_body.pdf and https://journals.plos.org/plosone/s/file?id=ba62/PLOSOne_formatting_sample_title_authors_affiliations.pdf

2. We note that your Data Availability Statement is currently as follows: All relevant data are within the manuscript and its Supporting Information files.

Please confirm at this time whether or not your submission contains all raw data required to replicate the results of your study. Authors must share the “minimal data set” for their submission. PLOS defines the minimal data set to consist of the data required to replicate all study findings reported in the article, as well as related metadata and methods (https://journals.plos.org/plosone/s/data-availability#loc-minimal-data-set-definition).

For example, authors should submit the following data:

- The values behind the means, standard deviations and other measures reported;

- The values used to build graphs;

- The points extracted from images for analysis.

Authors do not need to submit their entire data set if only a portion of the data was used in the reported study.

If your submission does not contain these data, please either upload them as Supporting Information files or deposit them to a stable, public repository and provide us with the relevant URLs, DOIs, or accession numbers. For a list of recommended repositories, please see https://journals.plos.org/plosone/s/recommended-repositories.

If there are ethical or legal restrictions on sharing a de-identified data set, please explain them in detail (e.g., data contain potentially sensitive information, data are owned by a third-party organization, etc.) and who has imposed them (e.g., an ethics committee). Please also provide contact information for a data access committee, ethics committee, or other institutional body to which data requests may be sent. If data are owned by a third party, please indicate how others may request data access.

[Note: HTML markup is below. Please do not edit.]

Reviewers' comments:

Reviewer's Responses to Questions

**Comments to the Author**

1. Is the manuscript technically sound, and do the data support the conclusions?

The manuscript must describe a technically sound piece of scientific research with data that supports the conclusions. Experiments must have been conducted rigorously, with appropriate controls, replication, and sample sizes. The conclusions must be drawn appropriately based on the data presented.

Reviewer #1: Yes

Reviewer #2: No

**********

2. Has the statistical analysis been performed appropriately and rigorously?

Reviewer #1: Yes

Reviewer #2: No

**********

3. Have the authors made all data underlying the findings in their manuscript fully available?

The PLOS Data policy requires authors to make all data underlying the findings described in their manuscript fully available without restriction, with rare exception (please refer to the Data Availability Statement in the manuscript PDF file). The data should be provided as part of the manuscript or its supporting information, or deposited to a public repository. For example, in addition to summary statistics, the data points behind means, medians and variance measures should be available. If there are restrictions on publicly sharing data—e.g. participant privacy or use of data from a third party—those must be specified.

Reviewer #1: Yes

Reviewer #2: No

**********

4. Is the manuscript presented in an intelligible fashion and written in standard English?

PLOS ONE does not copyedit accepted manuscripts, so the language in submitted articles must be clear, correct, and unambiguous. Any typographical or grammatical errors should be corrected at revision, so please note any specific errors here.

Reviewer #1: Yes

Reviewer #2: Yes

**********

5. Review Comments to the Author

Please use the space provided to explain your answers to the questions above. You may also include additional comments for the author, including concerns about dual publication, research ethics, or publication ethics. (Please upload your review as an attachment if it exceeds 20,000 characters)

Reviewer #1: This is an interesting study. Although I am not close to the subject, I could follow the ms and I think that the rat model and the system that the authors are developing can be useful. I have no suggestions for changes.

Reviewer #2: The authors attempted to re-create a model to study LAS using various anoxia and hypoxia controlled procedures. However, the authors did not demonstrate the necessary phenotypic responses often observed in human patients nor were they able to replicate previously established results.

Comments/questions:

What current treatments are available?

The weight and the sex of the animal can drastically affect oxygen tolerance and the subsequent downstream physiological and molecular responses. Is there a reason why only male rats were used?

Why wasn't the same anesthetic regiment used as hypoxia non-intubated animals? Extended exposure to isoflurane has significant effects on heart rate, which is an important parameter measured in your study. The authors must clearly demonstrate these differences and solidly prove that the reduced cardiac function is not due to extended isoflurane exposure.

What parameters were used to determine the number of animals for each study? Please elaborate on the power analysis performed to make these determinations.

There are molecular differences between hypoxia/anoxia and ischemia. Can the authors elaborate on what's already know in literature? It would be useful to measure these factors (ie. blood cytokines, some histological analysis on tissues), to establish a strong baseline on whether your model can recapitulate what happens during LAS in humans. These molecular changes are necessary to confirm whether the model (to be created) causes similar pathologies to humans.

Figure need to be uploaded with higher quality. Figure 5 was not legible at all.

**********

6. PLOS authors have the option to publish the peer review history of their article (what does this mean?). If published, this will include your full peer review and any attached files.

If you choose “no”, your identity will remain anonymous but your review may still be made public.

**Do you want your identity to be public for this peer review?**  For information about this choice, including consent withdrawal, please see our Privacy Policy.

Reviewer #1: No

Reviewer #2: No

**********

[NOTE: If reviewer comments were submitted as an attachment file, they will be attached to this email and accessible via the submission site. Please log into your account, locate the manuscript record, and check for the action link "View Attachments". If this link does not appear, there are no attachment files.]

While revising your submission, please upload your figure files to the Preflight Analysis and Conversion Engine (PACE) digital diagnostic tool, https://pacev2.apexcovantage.com/. PACE helps ensure that figures meet PLOS requirements. To use PACE, you must first register as a user. Registration is free. Then, login and navigate to the UPLOAD tab, where you will find detailed instructions on how to use the tool. If you encounter any issues or have any questions when using PACE, please email PLOS at figures@plos.org. Please note that Supporting Information files do not need this step.

# Author comment: Author response to Decision Letter 1

**DOI:** 10.1371/journal.pone.0317638.r002

11 Jun 2025

Reviewer #1: This is an interesting study. Although I am not close to the subject, I could follow the ms and I think that the rat model and the system that the authors are developing can be useful. I have no suggestions for changes.

We thank the reviewer for this comment.

Reviewer #2: The authors attempted to re-create a model to study LAS using various anoxia and hypoxia controlled procedures. However, the authors did not demonstrate the necessary phenotypic responses often observed in human patients nor were they able to replicate previously established results.

Comments/questions:

What current treatments are available?

The current treatments for patients with Lance-Adams syndrome (LAS) are pharmacological anti-myoclonic treatments, mainly benzodiazepines and anti-seizure medications. As we reported in an up-to-date systematic review, the three most commonly used drugs in LAS patients were clonazepam, valproate, and levetiracetam (work under review in another journal). Moreover, in the cohort we recently published, we showed that levetiracetam, valproate, clonazepam, zonisamide, and perampanel showed better long-term retention rates (Vellieux et al., Neurology, 2024, DOI: 10.1212/WNL.0000000000209994).

Non-pharmacological approaches have also been reported in medication-refractory patients, including deep brain stimulation, especially in the globus pallidus internus, and iterative electroconvulsive therapy, as we previously reported in one of our patients (Vellieux et al., Brain Stimulation, 2023, DOI: 10.1016/j.brs.2023.03.004).

Regarding animal models of post-hypoxic myoclonus, myoclonic jerks in post-hypoxic rats decreased with valproate, clonazepam, 5-hydroxytryptophan (Truong et al., Movement Disorders, 1994, DOI: 10.1002/mds.870090214), piracetam, levetiracetam, brivaracetam (Tai and Truong, Journal of Neural Transmission, 2007, DOI: 10.1007/s00702-007-0788-3), and gabapentin (Kanthasamy et al., European Journal of Pharmacology, 1996, DOI: 10.1016/0014-2999(95)00741-5).

We added this point in the Introduction of the revised manuscript: “Several anti-myoclonic treatments are available for the patients, including pharmacological anti-seizure medications, such as clonazepam, valproate, and levetiracetam, and neuro-stimulation approaches in medication-refractory patients, such as deep brain stimulation or electroconvulsive therapy (2,4–6).”

The weight and the sex of the animal can drastically affect oxygen tolerance and the subsequent downstream physiological and molecular responses. Is there a reason why only male rats were used?

We chose male Sprague-Dawley rats weighing around 250-300 g because this type of animal was used in previous animal models of post-hypoxic myoclonus (Truong et al., Movement Disorders, 1994, DOI: 10.1002/mds.870090214; Truong et al., Movement Disorders, 2000, DOI: 10.1002/mds.870150706).

We added this point in the Discussion of the revised manuscript: “The two rodent models of LAS employed either pharmacological or mechanical ischemic procedures on male Sprague-Dawley rats weighing 250-300 g (10). […] We chose male Sprague-Dawley rats because they were previously employed for LAS models and CA rodent models (18). Young adult rats around 250-300 g were easy to handle, especially during intubation.”.

Why wasn't the same anesthetic regiment used as hypoxia non-intubated animals? Extended exposure to isoflurane has significant effects on heart rate, which is an important parameter measured in your study. The authors must clearly demonstrate these differences and solidly prove that the reduced cardiac function is not due to extended isoflurane exposure.

For the first group of hypoxic non-intubated animals, we could not use an inhalation sedation method with hypnotic gas because our homemade hypoxia chamber was not equipped with a closed gas circuit with a filtration system to collect the released gas. We thus chose to use an intraperitoneal injection with hypnotic drugs for sedation, which is a usual administration route in rodents, and also considering the excellent handling skills of our team. We used ketamine and xylazine, also aware of the possible xylazine-induced bradycardia. However, it represents a classical sedation protocol in rodents, already used in former animal models of post-hypoxic myoclonus (Truong et al., Movement Disorders, 1994, DOI: 10.1002/mds.870090214; Truong et al., Movement Disorders, 2000, DOI: 10.1002/mds.870150706).

For the second group of experiments in anoxic intubated animals, we took advantage of the animals’ ventilation circuit to use gas inhalation for sedation, especially isoflurane, which presents a moderate cardio-respiratory depression compared to xylazine. Gas anesthesia is usually safe because the sedation level can easily be controlled during maintenance, and anesthesia risks are usually very low. In most cardiorespiratory arrest animal models, anesthesia in small animals such as rodents is carried out using narcotic gases such as isoflurane (Yu et al., Frontiers in Neuroscience, 2023, DOI: 10.3389/fnins.2022.1087725). During our experiments, we first proceeded to sedation induction with 3% isoflurane in a dedicated induction chamber, then proceeded to sedation maintenance (after intubation) with 1.5% isoflurane. We then monitored the heart rate of our animals during the first few minutes before performing anoxia to ensure the absence of bradycardia. Moreover, in the control group, animals were also subjected to sedation with isoflurane for 10 minutes, and none of them showed bradycardia or unexpected death. Finally, some authors showed that the heart rate of Sprague-Dawley rats (12–16 weeks old and weighing 320±24 g) was relatively high and stable during sedation with isoflurane (induction 4% and maintenance 2% for 45 minutes) (Murakami et al., Biological & Pharmaceutical Bulletin, 2014, DOI: 10.1248/bpb.b14-00012).

We added this point in the Discussion of the revised manuscript: “We could not use an inhalation sedation with hypnotic gas because our homemade hypoxia chamber was not equipped with a closed gas circuit with a filtration system to collect the released gas. We therefore used an intraperitoneal injection with hypnotic drugs. […] In this second group of animals, we took advantage of the animals’ ventilation circuit to use gas inhalation for sedation, especially isoflurane, which presents a moderate cardio-respiratory depression compared to xylazine. Gas anesthesia is usually safe because the sedation level can easily be controlled during maintenance, and anesthesia risks are usually very low (21). In most cardiorespiratory arrest animal models, anesthesia in small animals such as rodents is carried out using narcotic gases such as isoflurane (18).”

What parameters were used to determine the number of animals for each study? Please elaborate on the power analysis performed to make these determinations.

First, it should be noted that there is no prevalence or incidence data regarding LAS in humans, especially the proportion of patients who suffered from chronic post-hypoxic myoclonus (i.e., LAS) after an anoxic/ischemic event. Moreover, in the previous experimental studies that developed an animal model of post-hypoxic myoclonus, the authors did not mention any precise data about the survival rate of the experiments and the proportion of animals that suffered from myoclonic jerks after global cerebral ischemia. Given this absence of both human epidemiological data and previous validated data from former animal models, it was currently not possible to perform a conventional sample size calculation based on statistical power analysis. There is indeed no available information regarding the expected prevalence, survival rate, or the probability of successfully inducing the target phenotype in animals. Therefore, this study was designed as an exploratory (pilot) investigation, whose primary objective was to assess the feasibility and reproducibility of the model.

To comply with the ethical principles of the 3Rs (Replacement, Reduction, and Refinement), we have chosen to limit the number of animals used in this initial phase and to adjust our experimental protocol based on the results obtained as the procedures progressed: (i) for non-intubated hypoxic rats: sedative dosage, oxygen concentration in the gas cylinder used, gas inflow rate into the chamber, and (ii) for intubated anoxic rats: continuous vs. intermittent anoxia, duration of anoxia.

Our objectives were thus to observe whether the pathological phenotype can be induced, to evaluate the onset, severity, and reproducibility of myoclonic jerks, to monitor survival and welfare-related endpoints, and to estimate the variability of biological responses for future power calculations.

In conclusion, this preliminary dataset was initially thought to allow for a rigorous statistical justification of sample size in subsequent confirmatory experiments to obtain meaningful exploratory data and minimize unnecessary animal use.

There are molecular differences between hypoxia/anoxia and ischemia. Can the authors elaborate on what's already know in literature? It would be useful to measure these factors (ie. blood cytokines, some histological analysis on tissues), to establish a strong baseline on whether your model can recapitulate what happens during LAS in humans. These molecular changes are necessary to confirm whether the model (to be created) causes similar pathologies to humans.

We agree with the reviewer that the biochemical and cellular mechanisms involved in neuronal damage caused by hypoxia/anoxia and ischemia may vary. This variability may lead to heterogeneous biochemical consequences in the micro-environment of neurons, especially in energy depletion, ion homeostasis, cell membrane integrity, oxidative stress, excitotoxicity, and cell death pathways (Lipton, Physiological Reviews, 1999, DOI: 10.1152/physrev.1999.79.4.1431). We can mention a few examples. Experimentally, hypoxia and ischemia produce different physiological responses in the brain. Ischemia is associated with a marked elevation in extracellular glutamate concentrations, and glutamate antagonists attenuate the neuropathologic injury. Hypoxia (PaO2 20 mmHg for 20 minutes), without ischemia, is neither associated with an elevation in extracellular glutamate concentrations nor with neuropathologic changes in rats, meaning that hypoxia without ischemia seems better tolerated than ischemia by the brain (Pearigen et al., Brain Research, 1996, DOI: 10.1016/0006-8993(96)00215-6). Moreover, gene expression is injury-specific as well. Although ischemia induces robust expression of the stress proteins HSP72 and HSP32, hypoxia alone induces neither gene product (Pearigen et al., Brain Research, 1996, DOI: 10.1016/0006-8993(96)00215-6; Bergeron et al., Developmental Brain Research, 1998, DOI: 10.1016/s0165-3806(97)00169-7). The absence of pathologic sequelae of hypoxia without ischemia has been described in humans as well (Gray and Horner, JAMA, 1970; Rie and Bernad, Neurology, 1980). The relative benignity of hypoxia without ischemia was also demonstrated in lightly anesthetized primates in a 3.2% oxygen with nitrogen atmosphere. Neuropathologic changes were rare, and when seen, occurred in arterial border zones only (de Courten-Myers et al., Stroke, 1985, DOI: 10.1161/01.str.16.6.1016). Similar results occurred in hypoxic but normotensive cats maintained with a PO2 of 17 mmHg for 25 minutes. All animals were normal clinically and neuropathologically on recovery (Auer and Mivamoto, Soc Neurosci Abstracts, 1995). However, LAS is associated with prolonged and profound anoxic-ischemic events leading to clinical symptoms, mainly action myoclonus, and to histopathological abnormalities, although nonspecific (Castaigne et al., Revue Neurologique, 1964; Masson et al., Revue Neurologique, 1975; De Léan et al., Advances in Neurology, 1986; Hauw et al., Advances in Neurology, 1986).

In our previously mentioned work, which is under review in another journal, we showed that two-thirds of patients with LAS reported in the literature suffered from a primary hypoxic respiratory event, leading or not to cardiac arrest and thus global cerebral ischemia. For this main reason, we tried to induce a novel animal model of chronic post-hypoxic myoclonus using pure hypoxia/anoxia, leading to or not to bradycardia and cardiac arrest in our animals.

We added this point in the Discussion of the revised manuscript: “Patients with LAS indeed mainly suffered from a primary hypoxic respiratory event (2). The biochemical and cellular mechanisms involved in neuronal damage caused by hypoxia/anoxia and ischemia may vary (13). This variability may lead to heterogeneous biochemical consequences in the micro-environment of neurons, for example, on extracellular glutamate concentrations (14), gene expression (14,15), or neuropathological changes (16,17).”

Figure need to be uploaded with higher quality. Figure 5 was not legible at all.

The figures embedded in the PDF manuscript are indeed of low quality. However, a high-resolution version of each figure is available via the download link located at the top-right corner of each figure.

Attachment Submitted filename: Response to Reviewers.docx

# Peer review: Decision Letter 1

**DOI:** 10.1371/journal.pone.0317638.r003

**Authors:** Marcelo Hermes-Lima, Marcelo Hermes-Lima

4 Sep 2025

PONE-D-25-00224R1

Towards new animal models of pure hypoxic Lance-Adams syndrome

PLOS ONE

Dear Dr. Navarro,

Thank you for submitting your manuscript to PLOS ONE. After careful consideration, we have decided that your revised manuscript does not meet our criteria for publication and must therefore be rejected.

**We made this decision based on the comments of referee #3. I agree with his observations.**

I am so sorry that we cannot be more positive on this occasion, but hope that you appreciate the reasons for this decision.

Kind regards,

Marcelo Hermes-Lima, PhD

Academic Editor

PLOS ONE

[Note: HTML markup is below. Please do not edit.]

Reviewers' comments:

Reviewer's Responses to Questions

**Comments to the Author**

1. If the authors have adequately addressed your comments raised in a previous round of review and you feel that this manuscript is now acceptable for publication, you may indicate that here to bypass the “Comments to the Author” section, enter your conflict of interest statement in the “Confidential to Editor” section, and submit your "Accept" recommendation.

Reviewer #3: (No Response)

**********

2. Is the manuscript technically sound, and do the data support the conclusions?

The manuscript must describe a technically sound piece of scientific research with data that supports the conclusions. Experiments must have been conducted rigorously, with appropriate controls, replication, and sample sizes. The conclusions must be drawn appropriately based on the data presented.

Reviewer #3: No

**********

3. Has the statistical analysis been performed appropriately and rigorously?

Reviewer #3: No

**********

4. Have the authors made all data underlying the findings in their manuscript fully available?

The PLOS Data policy requires authors to make all data underlying the findings described in their manuscript fully available without restriction, with rare exception (please refer to the Data Availability Statement in the manuscript PDF file). The data should be provided as part of the manuscript or its supporting information, or deposited to a public repository. For example, in addition to summary statistics, the data points behind means, medians and variance measures should be available. If there are restrictions on publicly sharing data—e.g. participant privacy or use of data from a third party—those must be specified.

Reviewer #3: Yes

**********

5. Is the manuscript presented in an intelligible fashion and written in standard English?

PLOS ONE does not copyedit accepted manuscripts, so the language in submitted articles must be clear, correct, and unambiguous. Any typographical or grammatical errors should be corrected at revision, so please note any specific errors here.

Reviewer #3: (No Response)

**********

6. Review Comments to the Author

Please use the space provided to explain your answers to the questions above. You may also include additional comments for the author, including concerns about dual publication, research ethics, or publication ethics. (Please upload your review as an attachment if it exceeds 20,000 characters)

**Reviewer #3:**  The manuscript is well-written and describes important exploratory attempts. The authors aimed to establish a reproducible animal model of Lance-Adams syndrome (LAS), but all hypoxic or anaerobic treatment regimens were unsuccessful in inducing a myoclonic phenotype. Thus, this study failed to establish a valid disease model, which limits its translational medical value. The high mortality rate of 26% to 50% among the protocols is not only ethically controversial, but also undermines the feasibility of the proposed model. This is contrary to the stated goal of developing “less invasive methods with lower mortality”. The use of only male Sprague-Dawley rats, although based on precedent, ignores sex differences in oxygen tolerance and neurophysiology. Sample size calculations were not performed, and the basis for an exploratory “pilot study” was weak in view of the high mortality rate and lack of phenotypes. Different anesthetic regimens were used in different groups, with confounding effects on cardiovascular responses. The rationale for terminating hypoxia at a specific heart rate threshold is not well documented.

**********

7. PLOS authors have the option to publish the peer review history of their article (what does this mean?). If published, this will include your full peer review and any attached files.

If you choose “no”, your identity will remain anonymous but your review may still be made public.

**Do you want your identity to be public for this peer review?**  For information about this choice, including consent withdrawal, please see our Privacy Policy.

Reviewer #3: No

**********

[NOTE: If reviewer comments were submitted as an attachment file, they will be attached to this email and accessible via the submission site. Please log into your account, locate the manuscript record, and check for the action link "View Attachments". If this link does not appear, there are no attachment files.]

- - - - -

For journal use only: PONEDEC3

# Author comment: Author response to Decision Letter 2

**DOI:** 10.1371/journal.pone.0317638.r004

4 Oct 2025

You will find in this document our responses (in blue) to the Reviewer #3’s comments (in black).

The manuscript is well-written and describes important exploratory attempts.

The authors aimed to establish a reproducible animal model of Lance-Adams syndrome (LAS), but all hypoxic or anaerobic treatment regimens were unsuccessful in inducing a myoclonic phenotype. Thus, this study failed to establish a valid disease model, which limits its translational medical value.

1) We would like to emphasize that the primary aim of our article was to share with the scientific community our « negative results », specifically to prevent other research groups from repeating the two experimental protocols we tested to establish a model of post-anoxic myoclonus, or Lance-Adams syndrome. Our manuscript was explicitly submitted under the category “Research Article – Negative Results.”

To clearly emphasize the objective of our report, we have now modified the title of our manuscript as: “Towards new animal models of pure hypoxic Lance-Adams syndrome: negative results”.

Publishing such data helps reduce unnecessary duplication of animal experiments, enhances transparency, and ultimately advances the search for more effective and ethically sound models of this devastating condition.

We are continuing to develop a model of Lance–Adams syndrome using more invasive strategies. It is important to share with the scientific community that the two protocols we rigorously applied were not sufficient to elicit post-anoxic myoclonus.

The high mortality rate of 26% to 50% among the protocols is not only ethically controversial, but also undermines the feasibility of the proposed model. This is contrary to the stated goal of developing “less invasive methods with lower mortality”.

2) We fully agree that both procedures failed to induce a reproducible phenotype and were associated with elevated mortality. However, contrary to Reviewer 3’s concerns, these protocols were not ethically controversial. Both were reviewed and approved by the local Ethics Committee Charles Darwin CEEACD/N°5 and by the French Ministry of Research (APAFIS approvals #35162-2022060716206563 and #43666-2023083015044880). Given the severity of human anoxic injuries and the urgent need for accurate preclinical models, the potential risks of these procedures were justified and carefully managed. Importantly, high mortality rates are unfortunately consistent with the clinical reality of severe anoxia in patients.

Data on the mortality has been removed from the Abstract.

The use of only male Sprague-Dawley rats, although based on precedent, ignores sex differences in oxygen tolerance and neurophysiology.

3) We agree with the reviewer that our choice to perform our experiments only in male Sprague-Dawley rats is debatable and that it may influence oxygen tolerance and subsequent physiopathological responses. However, our experiments were fully exploratory, only based on previous successful ischemic procedures of other teams (Truong et al., Movement Disorders, 1994, DOI: 10.1002/mds.870090214; Truong et al., Movement Disorders, 2000, DOI: 10.1002/mds.870150706). We took advantage of these successful procedures to reuse the same rat strain, age and sex. If we had succeeded in inducing the desired myoclonic phenotype in male rats, we would obviously have replicated our experiments on females.

This point has been added in the Discussion.

Sample size calculations were not performed, and the basis for an exploratory “pilot study” was weak in view of the high mortality rate and lack of phenotypes.

4) First, it should be noted that there is no prevalence or incidence data regarding LAS in humans, especially the proportion of patients who suffered from chronic post-hypoxic myoclonus (i.e., LAS) after an anoxic/ischemic event. Moreover, in the previous experimental studies that aimed to develop an animal model of post-hypoxic myoclonus, the authors did not mention any precise data about the survival rate of the experiments and the proportion of animals that suffered from myoclonic jerks after global cerebral ischemia. Given this absence of both human epidemiological data and previous validated data from former animal models, it was currently not possible to perform a conventional sample size calculation based on statistical power analysis. There is indeed no available information regarding the expected prevalence, survival rate, or the probability of successfully inducing the target phenotype in animals. Therefore, this study was designed as an exploratory (pilot) investigation, whose primary objective was to assess the feasibility and reproducibility of the model.

To comply with the ethical principles of the 3Rs (Replacement, Reduction, and Refinement), we have chosen to limit the number of animals used in this initial phase and to adjust our experimental protocol based on the results obtained as the procedures progressed: (i) for non-intubated hypoxic rats: sedative dosage, oxygen concentration in the gas cylinder used, gas inflow rate into the chamber, and (ii) for intubated anoxic rats: continuous vs. intermittent anoxia, duration of anoxia.

Our objectives were to observe whether the pathological phenotype can be induced, to evaluate the onset, severity, and reproducibility of myoclonic jerks, to monitor survival and welfare-related endpoints, and to estimate the variability of biological responses for future power calculations.

In conclusion, this preliminary dataset was initially thought to allow for a rigorous statistical justification of sample size in subsequent confirmatory experiments to obtain meaningful exploratory data and minimize unnecessary animal use.

Different anesthetic regimens were used in different groups, with confounding effects on cardiovascular responses. The rationale for terminating hypoxia at a specific heart rate threshold is not well documented.

5) For the first group of hypoxic non-intubated animals, we could not use an inhalation sedation method with hypnotic gas because our homemade hypoxia chamber was not equipped with a closed gas circuit with a filtration system to collect the released gas. We thus chose to use an intraperitoneal injection with hypnotic drugs for sedation, which is a usual administration route in rodents, and also considering the excellent handling skills of our team. We used ketamine and xylazine, also aware of the possible xylazine-induced bradycardia. However, it represents a classical sedation protocol in rodents, already used in former animal models of post-hypoxic myoclonus (Truong et al., Movement Disorders, 1994, DOI: 10.1002/mds.870090214; Truong et al., Movement Disorders, 2000, DOI: 10.1002/mds.870150706).

For the second group of experiments in anoxic intubated animals, we took advantage of the animals’ ventilation circuit to use gas inhalation for sedation, especially isoflurane, which presents a moderate cardio-respiratory depression compared to xylazine. Gas anesthesia is usually safe because the sedation level can easily be controlled during maintenance, and anesthesia risks are usually very low. In most cardiorespiratory arrest animal models, anesthesia in small animals such as rodents is carried out using narcotic gases such as isoflurane (Yu et al., Frontiers in Neuroscience, 2023, DOI: 10.3389/fnins.2022.1087725). During our experiments, we first proceeded to sedation induction with 3% isoflurane in a dedicated induction chamber, then proceeded to sedation maintenance (after intubation) with 1.5% isoflurane. We then monitored the heart rate of our animals during the first few minutes before performing anoxia to ensure the absence of bradycardia. Moreover, in the control group, animals were also subjected to sedation with isoflurane for 10 minutes, and none of them showed bradycardia or unexpected death. Finally, some authors showed that the heart rate of Sprague-Dawley rats (12–16 weeks old and weighing 320±24 g) was relatively high and stable during sedation with isoflurane (induction 4% and maintenance 2% for 45 minutes) (Murakami et al., Biological & Pharmaceutical Bulletin, 2014, DOI: 10.1248/bpb.b14-00012).

Attachment Submitted filename: Response to Reviewers PLOS ONEvn.docx

# Peer review: Decision Letter 2

**DOI:** 10.1371/journal.pone.0317638.r005

**Authors:** Marcelo Hermes-Lima, Marcelo Hermes-Lima, Yuqing Li

19 Nov 2025

PONE-D-25-00224R2

Towards new animal models of pure hypoxic Lance-Adams syndrome: negative results.

PLOS ONE

Dear Dr. Navarro,

Thank you for submitting your manuscript to PLOS ONE. After careful consideration, we feel that it has merit but does not fully meet PLOS ONE’s publication criteria as it currently stands. Therefore, we invite you to submit a revised version of the manuscript that addresses the points raised during the review process.

Please submit your revised manuscript by Jan 03 2026 11:59PM. If you will need more time than this to complete your revisions, please reply to this message or contact the journal office at plosone@plos.org. When you're ready to submit your revision, log on to https://www.editorialmanager.com/pone/ and select the 'Submissions Needing Revision' folder to locate your manuscript file.

Please include the following items when submitting your revised manuscript:

- A rebuttal letter that responds to each point raised by the academic editor and reviewer(s). You should upload this letter as a separate file labeled 'Response to Reviewers'.
- A marked-up copy of your manuscript that highlights changes made to the original version. You should upload this as a separate file labeled 'Revised Manuscript with Track Changes'.
- An unmarked version of your revised paper without tracked changes. You should upload this as a separate file labeled 'Manuscript'.

-->If you would like to make changes to your financial disclosure, please include your updated statement in your cover letter. Guidelines for resubmitting your figure files are available below the reviewer comments at the end of this letter.

If applicable, we recommend that you deposit your laboratory protocols in protocols.io to enhance the reproducibility of your results. Protocols.io assigns your protocol its own identifier (DOI) so that it can be cited independently in the future. For instructions see: https://journals.plos.org/plosone/s/submission-guidelines#loc-laboratory-protocols. Additionally, PLOS ONE offers an option for publishing peer-reviewed Lab Protocol articles, which describe protocols hosted on protocols.io. Read more information on sharing protocols at https://plos.org/protocols?utm_medium=editorial-email&utm_source=authorletters&utm_campaign=protocols.

We look forward to receiving your revised manuscript.

Kind regards,

Yuqing Li, Ph.D.

Academic Editor

PLOS ONE

Journal Requirements:

If the reviewer comments include a recommendation to cite specific previously published works, please review and evaluate these publications to determine whether they are relevant and should be cited. There is no requirement to cite these works unless the editor has indicated otherwise.

Please review your reference list to ensure that it is complete and correct. If you have cited papers that have been retracted, please include the rationale for doing so in the manuscript text, or remove these references and replace them with relevant current references. Any changes to the reference list should be mentioned in the rebuttal letter that accompanies your revised manuscript. If you need to cite a retracted article, indicate the article’s retracted status in the References list and also include a citation and full reference for the retraction notice.

Additional Editor Comments (if provided):

[Note: HTML markup is below. Please do not edit.]

Reviewers' comments:

Reviewer's Responses to Questions

**Comments to the Author**

1. If the authors have adequately addressed your comments raised in a previous round of review and you feel that this manuscript is now acceptable for publication, you may indicate that here to bypass the “Comments to the Author” section, enter your conflict of interest statement in the “Confidential to Editor” section, and submit your "Accept" recommendation.

Reviewer #4: (No Response)

Reviewer #5: (No Response)

**********

2. Is the manuscript technically sound, and do the data support the conclusions?

The manuscript must describe a technically sound piece of scientific research with data that supports the conclusions. Experiments must have been conducted rigorously, with appropriate controls, replication, and sample sizes. The conclusions must be drawn appropriately based on the data presented.

Reviewer #4: Partly

Reviewer #5: Yes

**********

3. Has the statistical analysis been performed appropriately and rigorously?

Reviewer #4: No

Reviewer #5: Yes

**********

4. Have the authors made all data underlying the findings in their manuscript fully available?

The PLOS Data policy requires authors to make all data underlying the findings described in their manuscript fully available without restriction, with rare exception (please refer to the Data Availability Statement in the manuscript PDF file). The data should be provided as part of the manuscript or its supporting information, or deposited to a public repository. For example, in addition to summary statistics, the data points behind means, medians and variance measures should be available. If there are restrictions on publicly sharing data—e.g. participant privacy or use of data from a third party—those must be specified.

Reviewer #4: No

Reviewer #5: Yes

**********

5. Is the manuscript presented in an intelligible fashion and written in standard English?

PLOS ONE does not copyedit accepted manuscripts, so the language in submitted articles must be clear, correct, and unambiguous. Any typographical or grammatical errors should be corrected at revision, so please note any specific errors here.

Reviewer #4: Yes

Reviewer #5: Yes

**********

6. Review Comments to the Author

Please use the space provided to explain your answers to the questions above. You may also include additional comments for the author, including concerns about dual publication, research ethics, or publication ethics. (Please upload your review as an attachment if it exceeds 20,000 characters)

Reviewer #4: This manuscript reports the behavioral effects of three approaches for inducing hypoxic injury in rats. The primary outcome is a myoclonus score, depicted in Figure 5. No effect of the hypoxic insults was detected. This reporting of a neutral result is always difficult, because of the high burden of proving that failure to detect something means that it is not present. It is clear that the insult was sufficiently severe, because it conveyed high mortality.

There is a need for an animal model of post-hypoxic myoclonus. Aside from the cited papers from the 1990's, there has not been any work with such a model. One aspect of myoclonus that this paper glosses over is that status myoclonus observed in the early time after hypoxia may be unrecoverable, but be part of the clinical spectrum that includes LAS (e.g. https://pubmed.ncbi.nlm.nih.gov/24286857/ and https://pubmed.ncbi.nlm.nih.gov/27351833/)

1. The statistical analysis is vague. A few tests are named, but the actual comparisons are poorly defined. The key outcome is Figure 5: a time series before and after an intervention of repeated measurements in individual animals. One simple analysis would be to model the score, Y~ A(time)+B(group)+C(group x time)+error, accounting for clustering within the animal. To simplify, time could even be pre/post intervention. In that sort of model, the primary comparison would be whether coefficients for group or group x time differ from zero. Another approach would be to collapse all the observations before and after the injury (ignoring sequential time), and compare scores pre/post, again accounting for repeated measures

2. If the result is neutral, then the results should be expressed in terms of what difference between groups was observed with a confidence interval on that difference. It is not sufficient to say that a p-value was < or > some threshold, since p-values are not measures of effect size and are influenced by number of observations and choice of test. For example, the results might read: "The mean daily score for sham animals was 10±3 and for anoxic animals was 15±4 with a mean difference of 5 (95% CI -4 to 10)." This form will allow future researchers to know how large of a difference has been reliably excluded by these data.

3. Figure 5. The values graphed are mean (SD), but this ordinal scale is bounded by zero (values cannot be negative). It would be more appropriate to graph median (IQR) for this type of score. This figure would be more clear if the y-axis was moved to the left: a dashed line could still separate pre/post or indicate the intervention day.

4. There are a few odd phrasings: line 136 "brutal 100% N2 ventilation" - the word "brutal" implies violence, and I think the authors might have meant "severe". Truly, no adjective is required since "100% N2 ventilation" speaks for itself. lines 276-7 "we observed that rats almost systematically

died" - "systematically" probably should be "universally" or "always". If this can be a number (90% died) it would be more informative.

5. The discussion should mention two possibilities that may prevent the success of this effort. First, post-hypoxic myoclonus might be a sequella of severe brain injury which would require post-hypoxia intensive care. These models with spontaneously-breathing rats may be unable to detect the myoclonus, whereas more heroic or prolonged ventilations of some of the animals who died in these models might have revealed myoclonus. Second, the rat has an agyric cortex with substantial differences from humans. It is possible that the myoclonus syndrome is a feature of primate or more gyrencephalic brains.

Reviewer #5: PLOS One – Towards new animal models of pure hypoxic Lance-Adams syndrome: Negative Results

As was pointed out in previous reviews, this manuscript describes three logical, but ineffective, strategies to induce post-hypoxic startle myoclonus in rats, either by ambient replacement of oxygen with nitrogen in an enclosed chamber or by two different intubated ventilation regimes with 100% nitrogen. The previous review pointed out that the translational value of the manuscript is limited by negative outcomes (i.e., no rat phenotype resulted). Technical and statistical considerations also were raised. Yet, I am not convinced that those arguments are relevant by themselves for deciding to reject this manuscript.

If PLOS is amenable to publishing negative outcomes, I would ask them to consider the manuscript more fully because there is significant value, which I explain below. In addition, I believe that the manuscript can be improved by the authors to further increase value to those interested in this field, if the authors would more deeply discuss the clinical and scientific implications of their negative findings.

1. I have performed dozens of cardiac arrest procedures in rats using the mechanical metal-hook procedure that the authors consider the gold-standard to produce post-hypoxic startle myoclonus (disseminated by Truong et al., which the authors cite). That procedure is tricky and requires significant experience of a skilled investigator to reliably produce startle myoclonus with minimum mortality. Also, as an investigator learns the procedure, there can be significant ramp-up mortality. Not noted in the manuscript, the procedure can induce neurological symptoms adding to startle myoclonus (ataxia, acute seizures, paraplegia in cases), for which some local institutional veterinarians, Animal Care and Use Committees, and administrators are predisposed to view skeptically, even though humans suffer immensely with a much worse condition. It is an obvious and a worthy goal to identify a rat procedure that might be less invasive, less impairing, and produces less mortality, but still produces a clinically-relevant myoclonus phenotype using the methods described in this manuscript.

To try to achieve that goal, we, and perhaps others, tested many closely identical procedures in this manuscript years ago, with the identical negative outcomes described. It would be very useful if anoxia sans acute cardiac arrest effectively produced startle myoclonus in rats. However, as systematically evidenced in this manuscript, and by my and others’ prior unpublished efforts, this is not true.

Thus, given that future investigators may travel down this path and repeat, yet again, the negative experiments described here, I recommend publishing this manuscript. This is especially so given one Reviewer’s dissatisfaction that there was a degree of mortality using the approaches described. I’m less concerned by that rat mortality (there is no indication that those rats were treated inhumanely; the experiments were performed according to guidelines and approvals), given that human morbidity and mortality is far greater and solutions to this magnitude of brain hypoxia in the clinic are in fact needed. Publishing this manuscript would help ensure future investigators to direct their effort and resources elsewhere, benefitting everyone.

2. I was unconvinced by a previous Reviewer’s point that the value of the study was lessened by the lack of power analysis. Obviously, this is a pilot and no estimates of variance could have informed the experiments up front. Performing the procedures on a defined number of rats without sign of obvious myoclonic effect is likely sufficient to justify concluding that the procedures do not produce startle myoclonus of a magnitude approaching following anoxic cardiac arrest, without having to further establish that a larger group size would be needed to statistically validate a possible effect that is not obvious visually. Startle myoclonus in rats is very visually obvious.

3. It is, in fact, valuable to know that mortality using this non-invasive anoxia procedure was high, while the incidence of startle myoclonus was zero or near zero. The former illustrates the procedures were sufficient to evoke severe pathophysiology, but not of the type that produces startle myoclonus. This further justifies publishing the manuscript, in my view. This point could be expounded upon further by the authors.

4. I believe that the manuscript would be improved and that its value would be increased immensely if the authors would rewrite their Discussion to address what they may believe are the differences in pathophysiology between anoxia alone vs. anoxic/ischemic cardiac arrest in inducing the behavioral phenotype. As it stands now, the Discussion just recapitulates the procedures and negative outcomes, which are covered earlier and do not add value. The authors have thought long and hard about LA syndrome as evidenced in their prior review paper. I’d like the authors to address what they believe is the difference in pathophysiology between nearly fatal anoxia vs. ischemic/anoxic cardiac arrest, only the latter of which produces the startle myoclonus phenotype in rats.

Of note, we published two lengthy papers showing ischemic/anoxic cardiac arrest requires a threshold range of cardiac arrest parameters that produce rat PHM phenotype and that the behavior has a brainstem generator (Welsh et al Myoclonus and Paroxysmal Dyskinesias, Advances in Neurology 89, p. 307 and 331), uncited information that may be valuable to the authors. In our lab, PHM virtually never occurred when blood pressure without heart beat was not immediately lowered and sustained to below 10 mmHg for at least 7-7.5 min during complete anoxia, but not more than 9-9.5 min after which mortality was near 100%. Our own experiments indicated the neuron degeneration process occurred over 48-72 hr post-recovery, due to enhanced synaptic activity driven possibly by an upregulation in ionic channel conductances in rhythm-generating neurons. For those reasons, it became clear to us that even gradual reductions in brain oxygen, perhaps even sustained at a level near a very low threshold, would not produce PHM in rats – as reported here. It would be good for the authors to provide quantitative comments on this issue on why that is true.

5. Finally, it would be helpful for the authors, given their clinical experience, to comment upon whether these rat efforts adequately model human post-hypoxic myoclonus. Does the exaggerated startle response have sufficient construct validity for human post-hypoxic myoclonus (in spite of absence of negative myoclonus and asterixis)? More importantly, based on the authors’ expertise, does the requirement of the rat’s startle myoclonus behavior for ischemic/anoxia lower (or increase?) its face validity to human post-hypoxic myoclonus? Is the rat perhaps not the best species for modeling human post-hypoxic myoclonus given these attributes, while retaining its value for basic science? If the authors would critically address those issues in the Discussion (rather than recapitulating methods), the value of this paper – despite the negative outcomes – would be greatly enhanced.

Minor: Abstract: Rewrite the abstract for clarity to draw parallels between the 2 “groups” and 3 “procedures.” For instance, line 22 describes 2 experimental groups: hypoxia by cage replacement and hypoxia by intubation. Later (line 34) it describes 3 procedures: hypoxia without intubation; continuous anoxia by intubation; intermittent anoxia by intubation. This is confusing. I would describe the experiment as having 3 experimental groups, each with its own experimental procedure.

**********

7. PLOS authors have the option to publish the peer review history of their article (what does this mean?). If published, this will include your full peer review and any attached files.

If you choose “no”, your identity will remain anonymous but your review may still be made public.

**Do you want your identity to be public for this peer review?**  For information about this choice, including consent withdrawal, please see our Privacy Policy.

Reviewer #4: No

Reviewer #5: No

**********

[NOTE: If reviewer comments were submitted as an attachment file, they will be attached to this email and accessible via the submission site. Please log into your account, locate the manuscript record, and check for the action link "View Attachments". If this link does not appear, there are no attachment files.]

To ensure your figures meet our technical requirements, please review our figure guidelines: https://journals.plos.org/plosone/s/figures

You may also use PLOS’s free figure tool, NAAS, to help you prepare publication quality figures: https://journals.plos.org/plosone/s/figures#loc-tools-for-figure-preparation.

NAAS will assess whether your figures meet our technical requirements by comparing each figure against our figure specifications.

# Author comment: Author response to Decision Letter 3

**DOI:** 10.1371/journal.pone.0317638.r006

22 Feb 2026

You will find in this document our responses (in blue) to the Reviewers’ comments (in black).

Reviewer #4: This manuscript reports the behavioral effects of three approaches for inducing hypoxic injury in rats. The primary outcome is a myoclonus score, depicted in Figure 5. No effect of the hypoxic insults was detected. This reporting of a neutral result is always difficult, because of the high burden of proving that failure to detect something means that it is not present. It is clear that the insult was sufficiently severe, because it conveyed high mortality.

There is a need for an animal model of post-hypoxic myoclonus. Aside from the cited papers from the 1990's, there has not been any work with such a model. One aspect of myoclonus that this paper glosses over is that status myoclonus observed in the early time after hypoxia may be unrecoverable, but be part of the clinical spectrum that includes LAS (e.g. https://pubmed.ncbi.nlm.nih.gov/24286857/ and https://pubmed.ncbi.nlm.nih.gov/27351833/)

We agree with the reviewer that our medical and scientific community does need an animal model for post-hypoxic myoclonus. This extremely disabling condition represents a burden for surviving patients and their relatives in a context of no efficient therapy. An animal model would be of great help to better understand the pathophysiology of this neurological disorder facing many unmet medical needs.

We also agree with the reviewer that Lance-Adams syndrome belongs to the broader category of post-hypoxic myoclonic syndromes and that it represents its ‘chronic’ form associated with a good prognosis in terms of mortality, contrary to the ‘acute’ form of post-hypoxic myoclonus which is associated with a very poor prognosis. In our recent review in Brain Communications (DOI: 10.1093/braincomms/fcaf329), we highlighted this continuum outlining that few patients who will further develop LAS may previously show myoclonus during coma, with a different semiology. The diagnosis of LAS is therefore challenging in comatose patients. Some authors define as ‘acute’ post-hypoxic myoclonus any patient with myoclonus’ onset during the first hours or days after the anoxic event. The prognosis is heterogeneous among these patients. However, some other patients with ‘acute’ post-hypoxic myoclonus may then develop Lance-Adams syndrome, for some defined as ‘chronic’ post-hypoxic myoclonus after regaining consciousness, portending a far better prognosis, even if they presented with generalized myoclonus in the comatose post-anoxic period.

Our goal was to develop an animal model of the chronic form of post-hypoxic myoclonus, i.e. Lance-Adams syndrome, occurring in survivors of anoxic brain insult. This animal model would be a great help to confirm (or rule out) our pathophysiologic hypothesis obtained from the study of our human cohort, recently published in Neurology (DOI: 10.1212/WNL.0000000000209994).

1. The statistical analysis is vague. A few tests are named, but the actual comparisons are poorly defined. The key outcome is Figure 5: a time series before and after an intervention of repeated measurements in individual animals. One simple analysis would be to model the score, Y~ A(time)+B(group)+C(group x time)+error, accounting for clustering within the animal. To simplify, time could even be pre/post intervention. In that sort of model, the primary comparison would be whether coefficients for group or group x time differ from zero. Another approach would be to collapse all the observations before and after the injury (ignoring sequential time), and compare scores pre/post, again accounting for repeated measures.

2. If the result is neutral, then the results should be expressed in terms of what difference between groups was observed with a confidence interval on that difference. It is not sufficient to say that a p-value was < or > some threshold, since p-values are not measures of effect size and are influenced by number of observations and choice of test. For example, the results might read: "The mean daily score for sham animals was 10±3 and for anoxic animals was 15±4 with a mean difference of 5 (95% CI -4 to 10)." This form will allow future researchers to know how large of a difference has been reliably excluded by these data.

We appreciate the reviewer’s suggestion to employ a linear mixed-effects model (LMM) to account for the repeated-measures structure of our data. Below, we detail the implementation, results, and robustness checks of this approach.

We fitted the following LMM to our data: score ~ time * group + (1 | rat_id)

• “score” = myoclonus score (primary outcome).

• “time” = days before and after hypoxic or anoxic protocol

• “group” = experimental condition controls vs experimental protocol (hypoxia, continuous anoxia or intermittent anoxia).

• “(1 | rat_id)” = random intercept for each animal to account for within-subject correlation.

Analyses were conducted in R (v 4.5.1) using the “lme4” (v 1.1.37), “lmerTest” (v3.1.3), and “emmeans” (v2.0.0) packages. Model convergence was confirmed via diagnostic plots (residuals vs. fitted values, Q-Q plots) and the absence of singularity warnings.

The model output is summarized in the Tables below.

1/ Hypoxia vs controls (Table 1)

Effect Estimate (SE) 95% CI df p-value Sig.

Time -1.8833 (0.3188) [-2.5118, -1.2547] 1 <0.001 ***

Group 0.2689 (5.3754) [-11.1293, 11.6672] 1 0.9607 ns

Time:group 0.523 (0.3659) [-0.1982, 1.2443] 1 0.1543 ns

For the comparison of hypoxia versus controls, we found a main effect of time (p<0.001), suggesting a significant modification of the myoclonus score over time. The statistical model did not show significant global difference between the hypoxic group and the control group. We also did not demonstrate any significant interaction between the time and the group.

2/ Continuous anoxia vs controls (Table 2)

Effect Estimate (SE) 95% CI df p-value Sig.

Time -0.1795 (0.065) [-0.3073, -0.0517] 1 <0.001 ***

Group 0.0729 (1.4842) [-2.9862, 3.132] 1 0.9612 ns

Time:group -0.1323 (0.0772) [-0.2841, 0.0195] 1 0.0874 ns

For the comparison of continuous anoxia versus controls, we found a main effect of time (p<0.001), suggesting a significant modification of the myoclonus score over time. The statistical model did not show significant global difference between the continuous anoxia group and the control group. We also did not demonstrate any significant interaction between the time and the group.

3/ Intermittent anoxia vs controls (Table 3)

Effect Estimate (SE) 95% CI df p-value Sig.

Time -0.0789 (0.0536) [-0.1843, 0.0265] 1 <0.001 ***

Group 1.7031 (2.1795) [-2.8483, 6.2546] 1 0.4439 ns

Time:group -0.098 (0.0656) [-0.227, 0.031] 1 0.1360 ns

For the comparison of intermittent anoxia versus controls, we found a main effect of time (p<0.001), suggesting a significant modification of the myoclonus score over time. The statistical model did not show significant global difference between the intermittent anoxia group and the control group. We also did not demonstrate any significant interaction between the time and the group.

We added all these data to the updated version of the manuscript, in the Material & Methods section as in the Results section.

3. Figure 5. The values graphed are mean (SD), but this ordinal scale is bounded by zero (values cannot be negative). It would be more appropriate to graph median (IQR) for this type of score. This figure would be more clear if the y-axis was moved to the left: a dashed line could still separate pre/post or indicate the intervention day.

As requested by the reviewer, we changed the values on the three plots of Figure 5. In the new version of the manuscript, you will find the median (error bar: interquartile) on a plot with y-axis moved to the left and a line at day 0 separating pre- and post-protocol.

4. There are a few odd phrasings: line 136 "brutal 100% N2 ventilation" - the word "brutal" implies violence, and I think the authors might have meant "severe". Truly, no adjective is required since "100% N2 ventilation" speaks for itself. lines 276-7 "we observed that rats almost systematically

died" - "systematically" probably should be "universally" or "always". If this can be a number (90% died) it would be more informative.

We agree with the reviewer and have deleted the words ‘brutal’ line 136 and ‘brutally’ line 133.

We agree with the reviewer and have specified that “we observed that 75% of rats died when the HR was < 140-150 beats/min…”.

5. The discussion should mention two possibilities that may prevent the success of this effort. First, post-hypoxic myoclonus might be a sequella of severe brain injury which would require post-hypoxia intensive care. These models with spontaneously-breathing rats may be unable to detect the myoclonus, whereas more heroic or prolonged ventilations of some of the animals who died in these models might have revealed myoclonus. Second, the rat has an agyric cortex with substantial differences from humans. It is possible that the myoclonus syndrome is a feature of primate or more gyrencephalic brains.

We thank the reviewer for these two important comments.

1/ The first hypothesis of the reviewer is that a more prolonged resuscitation of animals would bring more survivors and then more animals prone to developing post-hypoxic myoclonus. We agree with this comment. We had discussed at the end of our article some methods that would refine our manipulations: capnograph for capnography-guided procedures, intravascular arterial and venous catheters for invasive monitoring of arterial blood pressure and administration of catecholamines.

Moreover, we agree that post-anoxic resuscitation itself, including reoxygenation and mechanical ventilation parameters, may contribute to secondary brain injury and should therefore be considered in the pathophysiological interpretation of post-anoxic phenomena. Experimental and clinical studies have shown that hyperoxia following return of spontaneous circulation can exacerbate oxidative stress and neuroinflammation. In animal models of cardiac arrest, ventilation with 100% oxygen after resuscitation has been associated with worse neurological outcomes compared with normoxic strategies (Liu et al., 1998, Stroke). More recent experimental work has also demonstrated increased inflammatory and apoptotic signaling in the brain following hyperoxic resuscitation (Aoki et al., 2023, Scientific Reports). In parallel, clinical data suggest that extreme PaCO₂ values, particularly severe hypocapnia, are associated with poorer neurological outcomes after cardiac arrest (Okada et al., 2022, Journal of Clinical Medicine; Xue et al., 2025, Anaesthesia Critical Care & Pain Medicine). These findings support the concept that post-resuscitation management may modulate the extent of global cerebral injury through oxidative and cerebrovascular mechanisms.

Importantly, the current literature does not identify reoxygenation toxicity per se as a specific mechanistic trigger of post-hypoxic myoclonus. While oxidative stress and inflammation may contribute to the overall severity of neuronal damage, available evidence does not support a direct and selective role of hyperoxia or ventilator-induced effects in generating myoclonic activity. Rather, these factors appear to modulate injury severity at a global level.

2/ The smooth lissencephalic cortex of the rat differs markedly from the folded gyrencephalic cortex of humans and primates, which may impact the capacity of the rodent brain to exhibit complex network phenomena such as sustained involuntary movements after global hypoxic injury. These gyrification differences correlate with quantitative and qualitative distinctions in cortex organization. Non-human primates and humans exhibit a larger proportion of isocortex and expanded prefrontal regions than rodents, which are associated with higher-order motor control, complex sensory integration, and sustained cognitive processing not paralleled in rodent models (Zhang et al., 2023, National Science Review). Post-hypoxic myoclonus in humans, as a gyrencephalic species, may likely engage distributed cortical loops involving extensive sensorimotor territories, including motor, premotor and supplementary motor areas that are disproportionately expanded in gyrencephalic cortices (Vellieux et al. Neurology, 2024). The absence of sulci and extensive gyri in rats might limit the emergence or expression of certain network-level disturbances that underlie sustained myoclonic syndromes after diffuse hypoxic injury in humans. Therefore, while basic neuronal and synaptic processes are conserved across mammals, the topological and network-level differences associated with gyrification and cortical expansion should be acknowledged as limitations in modelling certain human clinical phenotypes using rodents.

However, some authors have reported results of myoclonic phenotypes in rodents through various contexts, for example as already specified post-hypoxic myoclonus (Truong et al., 2000, Movement Disorders), but also progressive myoclonic epilepsy (Buzzi et al., 2012, Neurobiology of Disease) or juvenile myoclonic epilepsy (Velisek et al., 2011, PLoS One; Arain et al., 2016, Neurobiology of Disease), outlining the fact that it seems realistic to try to create a myoclonic phenotype in rodents.

We added these two points of discussion to the dedicated section of the new version of the manuscript.

Reviewer #5: PLOS One – Towards new animal models of pure hypoxic Lance-Adams syndrome: Negative Results

As was pointed out in previous reviews, this manuscript describes three logical, but ineffective, strategies to induce post-hypoxic startle myoclonus in rats, either by ambient replacement of oxygen with nitrogen in an enclosed chamber or by two different intubated ventilation regimes with 100% nitrogen. The previous review pointed out that the translational value of the manuscript is limited by negative outcomes (i.e., no rat phenotype resulted). Technical and statistical considerations also were raised. Yet, I am not convinced that those arguments are relevant by themselves for deciding to reject this manuscript.

If PLOS is amenable to publishing negative outcomes, I would ask them to consider the manuscript more fully because there is significant value, which I explain below. In addition, I believe that the manuscript can be improved by the authors to further increase value to those interested in this field, if the authors would more deeply discuss the clinical and scientific implications of their negative findings.

1. I have performed dozens of cardiac arrest procedures in rats using the mechanical metal-hook procedure that the authors consider the gold-standard to produce post-hypoxic startle myoclonus (disseminated by Truong et al., which the authors cite). That procedure is tricky and requires significant experience of a skilled investigator to reliably produce startle myoclonus with minimum mortality. Also, as an investigator learns the procedure, there can be significant ramp-up mortality. Not noted in the manuscript, the procedure can induce neurological symptoms adding to startle myoclonus (ataxia, acute seizures, paraplegia in cases), for which some local institutional veterinarians, Animal Care and Use Committees, and administrators are predisposed to view skeptically, even though humans suffer immensely with a much worse condition. It is an obvious and a worthy goal to identify a rat procedure that might be less invasive, less impairing, and produces less mortality, but still produces a clinically-relevant myoclonus phenotype using the methods described in this manuscript.

To try to achieve that goal, we, and perhaps others, tested many closely identical procedures in this manuscript years ago, with the identical negative outcomes described. It would be very useful if anoxia sans acute cardiac arrest effectively produced startle myoclonus in rats. However, as systematically evidenced in this manuscript, and by my and others’ prior unpublished efforts, this is not true.

Thus, given that future investigators may travel down this path and repeat, yet again, the negative experiments described here, I recommend publishing this manuscript. This is especially so given one Reviewer’s dissat

Attachment Submitted filename: Response_to_Reviewers_auresp_3.docx

# Peer review: Decision Letter 3

**DOI:** 10.1371/journal.pone.0317638.r007

**Authors:** Marcelo Hermes-Lima, Marcelo Hermes-Lima, Yuqing Li, Yuqing Li

9 Mar 2026

<div>PONE-D-25-00224R3

Towards new animal models of pure hypoxic Lance-Adams syndrome: negative results.

PLOS One

Dear Dr. Navarro,

Thank you for submitting your manuscript to PLOS ONE. After careful consideration, we feel that it has merit but does not fully meet PLOS ONE’s publication criteria as it currently stands. Therefore, we invite you to submit a revised version of the manuscript that addresses the points raised during the review process.

Please submit your revised manuscript by Apr 23 2026 11:59PM. If you will need more time than this to complete your revisions, please reply to this message or contact the journal office at plosone@plos.org. When you're ready to submit your revision, log on to https://www.editorialmanager.com/pone/ and select the 'Submissions Needing Revision' folder to locate your manuscript file.

Please include the following items when submitting your revised manuscript:

- A letter that responds to each point raised by the academic editor and reviewer(s). You should upload this letter as a separate file labeled 'Response to Reviewers'.
- A marked-up copy of your manuscript that highlights changes made to the original version. You should upload this as a separate file labeled 'Revised Manuscript with Track Changes'.
- An unmarked version of your revised paper without tracked changes. You should upload this as a separate file labeled 'Manuscript'.

-->If you would like to make changes to your financial disclosure, please include your updated statement in your cover letter. Guidelines for resubmitting your figure files are available below the reviewer comments at the end of this letter.

If applicable, we recommend that you deposit your laboratory protocols in protocols.io to enhance the reproducibility of your results. Protocols.io assigns your protocol its own identifier (DOI) so that it can be cited independently in the future. For instructions see: https://journals.plos.org/plosone/s/submission-guidelines#loc-laboratory-protocols. Additionally, PLOS ONE offers an option for publishing peer-reviewed Lab Protocol articles, which describe protocols hosted on protocols.io. Read more information on sharing protocols at https://plos.org/protocols?utm_medium=editorial-email&utm_source=authorletters&utm_campaign=protocols.

We look forward to receiving your revised manuscript.

Kind regards,

Yuqing Li, Ph.D.

Academic Editor

PLOS One

Journal Requirements:

If the reviewer comments include a recommendation to cite specific previously published works, please review and evaluate these publications to determine whether they are relevant and should be cited. There is no requirement to cite these works unless the editor has indicated otherwise.

Please review your reference list to ensure that it is complete and correct. If you have cited papers that have been retracted, please include the rationale for doing so in the manuscript text, or remove these references and replace them with relevant current references. Any changes to the reference list should be mentioned in the rebuttal letter that accompanies your revised manuscript. If you need to cite a retracted article, indicate the article’s retracted status in the References list and also include a citation and full reference for the retraction notice.

[Note: HTML markup is below. Please do not edit.]

Reviewers' comments:

Reviewer's Responses to Questions

**Comments to the Author**

1. If the authors have adequately addressed your comments raised in a previous round of review and you feel that this manuscript is now acceptable for publication, you may indicate that here to bypass the “Comments to the Author” section, enter your conflict of interest statement in the “Confidential to Editor” section, and submit your "Accept" recommendation.

Reviewer #4: All comments have been addressed

Reviewer #5: All comments have been addressed

**********

2. Is the manuscript technically sound, and do the data support the conclusions?

The manuscript must describe a technically sound piece of scientific research with data that supports the conclusions. Experiments must have been conducted rigorously, with appropriate controls, replication, and sample sizes. The conclusions must be drawn appropriately based on the data presented.

Reviewer #4: Yes

Reviewer #5: Partly

**********

3. Has the statistical analysis been performed appropriately and rigorously?

Reviewer #4: Yes

Reviewer #5: Yes

**********

4. Have the authors made all data underlying the findings in their manuscript fully available?

The PLOS Data policy requires authors to make all data underlying the findings described in their manuscript fully available without restriction, with rare exception (please refer to the Data Availability Statement in the manuscript PDF file). The data should be provided as part of the manuscript or its supporting information, or deposited to a public repository. For example, in addition to summary statistics, the data points behind means, medians and variance measures should be available. If there are restrictions on publicly sharing data—e.g. participant privacy or use of data from a third party—those must be specified.

Reviewer #4: Yes

Reviewer #5: Yes

**********

5. Is the manuscript presented in an intelligible fashion and written in standard English?

PLOS ONE does not copyedit accepted manuscripts, so the language in submitted articles must be clear, correct, and unambiguous. Any typographical or grammatical errors should be corrected at revision, so please note any specific errors here.

Reviewer #4: Yes

Reviewer #5: No

**********

6. Review Comments to the Author

Please use the space provided to explain your answers to the questions above. You may also include additional comments for the author, including concerns about dual publication, research ethics, or publication ethics. (Please upload your review as an attachment if it exceeds 20,000 characters)

Reviewer #4: The authors have addressed all of my suggestions very well. The results are a useful contribution to the literature. Figure 5 is OK, but still might benefit from larger fonts on axis labels, and suppressing the negative part of the y-axis (since the score can never be negative). I also would have nudged one of the groups to the left or right so the error bars do not overlap. This is purely an artistic suggestion.

Reviewer #5:

My review is uploaded as an attachment.

**********

7. PLOS authors have the option to publish the peer review history of their article (what does this mean?). If published, this will include your full peer review and any attached files.

If you choose “no”, your identity will remain anonymous but your review may still be made public.

**Do you want your identity to be public for this peer review?**  For information about this choice, including consent withdrawal, please see our Privacy Policy.

Reviewer #4: No

Reviewer #5: No

**********

[NOTE: If reviewer comments were submitted as an attachment file, they will be attached to this email and accessible via the submission site. Please log into your account, locate the manuscript record, and check for the action link "View Attachments". If this link does not appear, there are no attachment files.]

To ensure your figures meet our technical requirements, please review our figure guidelines: https://journals.plos.org/plosone/s/figures

You may also use PLOS’s free figure tool, NAAS, to help you prepare publication quality figures: https://journals.plos.org/plosone/s/figures#loc-tools-for-figure-preparation.

NAAS will assess whether your figures meet our technical requirements by comparing each figure against our figure specifications.

Attachment Submitted filename: Comments on myoclonus paper.pdf

# Author comment: Author response to Decision Letter 4

**DOI:** 10.1371/journal.pone.0317638.r008

22 Apr 2026

You will find in this document our responses (in blue) to the Reviewers’ comments (in black).

Reviewer #4

The authors have addressed all of my suggestions very well. The results are a useful contribution to the literature. Figure 5 is OK, but still might benefit from larger fonts on axis labels, and suppressing the negative part of the y-axis (since the score can never be negative). I also would have nudged one of the groups to the left or right so the error bars do not overlap. This is purely an artistic suggestion.

We thank the reviewer for this overall comment about the new version of our manuscript.

We could not modify the figure 5 due to a problem with our previous software.

Reviewer #5

I continue to support publishing this manuscript for the reasons I stated in my prior review, which remain in full effect and won’t be described further. The authors responded to my prior comments by editing the manuscript and it is greatly improved in places. However, I believe that it must undergo further editing before publication, so that the experimental procedures and results are transparent to investigators that may want to improve upon this study. The suggested edits below are not meant to indicate reduced enthusiasm for the study, but rather are meant to enhance its organization, precision, and language in order to enhance its impact. I acknowledge that the awkward use of English at times may be due to the authors being native French writers.

1. Reorganize and clearly state the experimental groups to be consistent with the revised Abstract.

The authors must restructure their experimental groups to be consistent with the Abstract. Currently, it is very difficult to piece together how the various groups differed from one another along the many dimensions in which they varied, as this information is highly fragmented among the Methods, Results, and Figure Legends. Obviously, it is essential to structure the experimental groups identically in the Abstract and the body of the paper.

To be helpful, I took the liberty of assembling a Table of Experimental Procedures and Groups based on what was written. I very strongly suggest that the authors include this Table in the main body of the paper and restructure their Methods and Results sections to match the Table (edit and include missing information highlighted in yellow). Please edit all other manuscript sections to be organized with regard to this this table. Also, reduce redundancy. As examples, the Discussion may need to be edited to match the experimental groups in certain places; the figures may need to be edited to reflect those groups; information about independent variables in each of the groups may need to be recalculated and moved from the Results to the Methods sections (i.e., mean anoxia duration, etc.). Note: I recognize that some of the information in the Table is in Figure 3, but I find Figure 3 to fall significantly short of the experimental detail that needs to be fully transparent in order to achieve the real purpose of this paper which is to alert investigators not to reproduce methods that do not produce a PHM phenotype in rats.

It is important to emphasize that there are 3 experimental groups and 2 control groups in the myoclonus experiment, with total n=59 for the myoclonus experiments from a total of 105 rats.

As requested by the reviewer, we created a Table 1 with all our experimental results and added it to the new version of our manuscript.

We also renamed throughout the manuscript the experimental groups as requested, i.e. Group 1, Group 2, Group 3, Group 1 controls, and Groups 2 and 3 controls.

2. Comparison to prior studies of mechanical whole-brain ischemia

It is no surprise that rats with strong hearts perhaps exceeding 60 bpm through a bout of hypoxia or anoxia did not exhibit PHM. In our experiments with the mechanical approach, PHM never resulted if perfusion was not reduced to near zero in a few seconds and maintained there for at least 7 min with anoxia and near 0 mmHg blood pressure (shock) for that duration. Note, in perhaps all of our PHM cases, a greatly weakened heart was itself already reduced to near 0 bpm by 5 min of mechanical ischemia that was sustained for the subsequent duration of anoxia (7-10 min total) before resuscitation.

Also in our and others’ experience, which the authors could have deduced from carefully reading the mechanical ischemia PHM literature, short-acting ketamine anesthesia was used alone - without xylazine, because the addition of xylazine as a long-duration CNS depressant and as an adjuvant to prolong ketamine’s action invariably prevented surviving prolonged anoxia/ischemia (100% mortality).

Thus, in the present experiments, the investigator’s use of xylazine explains the 100% mortality in the Pilot Study (see Table) in which the threshold for resuscitation was severe (complete and sustained respiratory suppression). The subsequent use of a far less pathological threshold (140-150 bpm) as a reaction to the Pilot Study mortality (even ensured by pre-anoxia epinephrine in Group 2!), would certainly prevent the PHM phenotype in any rat surviving those procedures. It can be inferred, therefore, that Group 1-3 non-survivors did not die of the pathophysiology that produces PHM, but of something different (but see below re: the variable threshold). Inserting the requested data in the new Table (yellow highlights, averages and ranges) will help readers understand whether other factors may have contributed (i.e., greater hypoxia/anoxia duration, lesser cage PO2, etc.).

We agree that all of the available sedative drugs, used alone or in combination, have side effects. However, the combination of ketamine and xylazine, as a classical anesthetic procedure, was requested by ethical committees and animal facilities. By consequence, this combination may lead to increased mortality, an issue we faced during our experiments. We have now added this important point to the Discussion, emphasizing that xylazine should be avoided in future experiments.

Moreover, as requested by the reviewer, we added all the experimental data in the new Table 1.

3. Resuscitation threshold.

Regarding the heart rate threshold for resuscitating Groups 1-3, the authors should describe this precisely. The threshold is described as “below 140-150 bpm,” which is vague. How far below (surely not 0 bpm, but 10?, 100?, 145?)? Why describe the threshold as a range? Do the authors, instead, mean that the threshold was between 140 and 149 bpm? Nearly that? The threshold should be clarified and edited everywhere it is described. How variable were the minimum heart rates across rats? Add threshold data to the Table if you have it (average and range across rats for survivors and non-survivors), as this may be clarifying (perhaps non-survivors had lower heart rates) as help to future investigators. As a placeholder, the new Table shows ≤ 140-150 bpm, but that needs to be edited too.

To clarify, we specified throughout the manuscript that procedures were stopped when heart rate was lower to 150 bpm.

4. Control groups.

It is remarkably incorrect to write that previous studies of rat startle PHM did not use pre-anoxia comparisons or comparisons to control animals (lines 79-81). The authors should carefully read Truong et al (2000) Movement Disorders, p. 26, Welsh et al (2002) Myoclonus and Paroxysmal Dyskinesias, Advances in Neurology, p. 307, and Welsh et al (2002) ibid, p. 331 in which both controls were used. Remarkably, the authors cited Truong et al (2000), indicating they did not read it. And, I am concerned that the authors did not read the two Welsh et al (2002) papers which were provided in my previous review. The authors should carefully read all 3 of those papers, revise their text based on their reading and citing of them, and consider them more carefully with regard to Point 2 and in consideration of their mechanistic explanations in the Discussion. By scholarly reading those papers and others that followed them, the authors will find yet more instances of the use of appropriate control groups, if they care to.

We are sorry for these errors. We corrected our manuscript and deleted the fact that previous animal models were performed without comparison to control animals.

5. Non-standardized oxygen concentration (0, 2, 4, and 6%) in Group 1 (lines 116-118).

The authors should explain how this was handled in their experimental design and specify it in the new Table. It is unclear why oxygen was varied and when the authors chose to do this. Please explain. Introducing this as an unaccounted-for variable is concerning. How did that relate to mortality?

With procedures on sedated non-intubated rats subjected to hypoxia in a dedicated cage (Group 1), we aimed to create different experimental conditions to explore the parameters that could generate the desired myoclonic phenotype. To this end, we introduced variability into our experimental conditions by using, on the one hand, oxygen/nitrogen cylinders with different concentration ratios (mainly to vary the minimal partial pressure of oxygen at equilibrium during the procedure) and, on the other hand, a flowmeter to vary the gas inflow rate into the hypoxia chamber (mainly to vary the slope of decrease in the partial pressure of oxygen inside the cage during the procedure).

We specified these arguments in our manuscript and the number of rats that received ventilation in the hypoxia chamber with cylinders containing 0%, 2%, 4%, or 6% oxygen in Table 1.

Language and Other Edits

6. The phrase “rats benefitted from the myoclonus assessment” (or its converse “rats that did not benefit from the myoclonus assessment”) used throughout the manuscript is incorrect and must be changed everywhere “benefitted” is used (note: no rat benefitted from any aspect of these experiments; all experimenters benefitted). I suggest the phrase “rats that were studied using the myoclonus assessment” or some proper English variant thereof.

We changed the sentences as requested, from “benefited from…” to “were studied with…”.

7. The phrase “systematically died” is awkward and morbid. According to what system did they die, and what evidence was collected to reveal that system? I suggest removing the adverb “systematically,” unless the authors are trying to make a physiological point in which case it should be explained. As for all suggested edits, this should be applied globally throughout the manuscript.

We removed the word “systematically” as requested.

8. Line 166: “At the first signs of awakening, the rats were progressively weaned from the ventilator and extubated.” In my experience, weaning from the ventilator begins when spontaneous breaths first begin to fight the ventilator. That is not synonymous with awakening, as most rats are in coma at this stage (see the literature). In my experience, some rats post severe anoxia will begin to breathe spontaneously and can be weaned from the ventilator, but never awaken. Clarify.

We totally agree with the reviewer and clarified the sentence as follows: “As soon as spontaneous ventilation resumed, the rats…”.

9. Line 172: “We systematically administered an epinephrine injection…” Remove the adverb “systematically,” or describe your “system” if it is something more important than filling a syringe.

We removed the word “systematically” as requested.

10. Line 178: why not say “intermittent hypoxia,” since that is the defining term of this section?

We replaced the word “discontinuous” to “intermittent” as requested.

11. Line 183: Begin paragraph with “Control rats (n=6) were…” or “To serve as no-hypoxia controls for Groups 2 and 3,…”

We changed the sentence as requested.

12. Line 246: “After the hypoxia procedure”: How long after the hypoxia procedure?

We clarified the sentences throughout the manuscript as follows: “We did not observe visible spontaneous and wandering-induced abnormal muscular jerks in freely moving post-hypoxic/anoxic rats up to X days after the hypoxia/anoxia procedure.”

13. Line 317: new paragraph when discussing Group 3. In general, good scientific writing begins the results of each experimental group as a new paragraph.

We added a new paragraph as requested.

14. Line 318: restating mortality is unnecessary.

As this mortality rate is specific for this group, we chose to precise this data.

15. I don’t know if this is a journal issue or an author issue, but it is highly distracting and prevents easy reading to have the figure captions inserted into the body of the document without spacing and separated far from the figure. Why aren’t the legends with the figures?

We totally agree with the reviewer, but as requested by the Instructions for authors of PLOSOne (https://journals.plos.org/plosone/s/submission-guidelines): “Figure captions must be inserted in the text of the manuscript, immediately following the paragraph in which the figure is first cited (read order). Do not include captions as part of the figure files themselves or submit them in a separate document.”

16. Figures: All figures should clearly refer to Groups 1, 2 or 3.

We specified Groups 1, 2 and 3 on updated figures and captions.

Discussion

17. Line 370: replace “8 to 9.5 min” to “7 to 10.5 min.” Read and cite Welsh et al (2002), Adv Neurol, Chapter 32 to see quantitative description of this.

As requested by the reviewer, we replaced this data in our manuscript and cited the reported reference.

18. Line 373-375. The intensity of the myoclonic jerk in the mechanical cardiac arrest model of PHM does not peak at day 4 post surgery. The best quantitative measures of the PHM audiogenic jerk by capacitive sensors and multi-muscle EMG indicate that it is most severe 2 days post anoxia/ischemia, and decreases nearly linearly over the following 3 weeks. Read and cite Welsh et al (2002), Adv Neurol, Chapter 31 to see high-res, quantitative analyses that were designed to be more sensitive than the myoclonus score.

As requested by the reviewer, we specified this data in our manuscript and cited the reported reference.

19. Lines 376-377. Rats having undergone mechanical cardiac arrest leading to PHM are often very ataxic for weeks following anoxia, perhaps due to action-induced myoclonic jerks. Although that was not well studied, those rats show slower and jerkier voluntary (i.e. conditioned) arm movements during the period that they show audiogenic myoclonus (see and cite Welsh et al (2002) Adv Neurol, Chapter 31). To a degree, those behavioral observations counter the authors’ suggestion here that the mechanical cardiac arrest model lacks face validity to human PHM because it induces audiogenic jerks only. This should be stated.

As requested by the reviewer, we specified this data in our manuscript and cited the reported reference.

20. Line 389. See Welsh et al (2002) Adv Neurol Chapter 32 and a growing clinical literature about cerebellar involvement in PHM as a complement to M1 involvement. EMG data suggests that rat cardiac arrest PHM is driven by a brainstem generator (see Welsh et al Adv Neurol Chapter 31).

As requested by the reviewer, we specified this possible involvement of subcortical structures in posthypoxic myoclonus.

21. Line 441. Replace “note” with “not.”

Typo corrected.

22. The Discussion is greatly improved from the previous version and appreciated.

We thank the reviewer for this comment.

Attachment Submitted filename: Response to Reviewers-final.docx

# Peer review: Decision Letter 4

**DOI:** 10.1371/journal.pone.0317638.r009

**Authors:** Marcelo Hermes-Lima, Marcelo Hermes-Lima, Yuqing Li, Yuqing Li, Yuqing Li

4 May 2026

Towards new animal models of pure hypoxic Lance-Adams syndrome: negative results.

PONE-D-25-00224R4

Dear Dr. Navarro,

We’re pleased to inform you that your manuscript has been judged scientifically suitable for publication and will be formally accepted for publication once it meets all outstanding technical requirements.

Within one week, you’ll receive an e-mail detailing the required amendments. When these have been addressed, you’ll receive a formal acceptance letter and your manuscript will be scheduled for publication.

An invoice will be generated when your article is formally accepted. Please note, if your institution has a publishing partnership with PLOS and your article meets the relevant criteria, all or part of your publication costs will be covered. Please make sure your user information is up-to-date by logging into Editorial Manager at Editorial Manager® and clicking the ‘Update My Information' link at the top of the page. For questions related to billing, please contact billing support.

If your institution or institutions have a press office, please notify them about your upcoming paper to help maximize its impact. If they’ll be preparing press materials, please inform our press team as soon as possible -- no later than 48 hours after receiving the formal acceptance. Your manuscript will remain under strict press embargo until 2 pm Eastern Time on the date of publication. For more information, please contact onepress@plos.org.

Kind regards,

Yuqing Li, Ph.D.

Academic Editor

PLOS One

Additional Editor Comments (optional):

Reviewers' comments:

Reviewer's Responses to Questions

**Comments to the Author**

1. If the authors have adequately addressed your comments raised in a previous round of review and you feel that this manuscript is now acceptable for publication, you may indicate that here to bypass the “Comments to the Author” section, enter your conflict of interest statement in the “Confidential to Editor” section, and submit your "Accept" recommendation.

Reviewer #5: All comments have been addressed

**********

2. Is the manuscript technically sound, and do the data support the conclusions?

The manuscript must describe a technically sound piece of scientific research with data that supports the conclusions. Experiments must have been conducted rigorously, with appropriate controls, replication, and sample sizes. The conclusions must be drawn appropriately based on the data presented.

Reviewer #5: Yes

**********

3. Has the statistical analysis been performed appropriately and rigorously?

Reviewer #5: Yes

**********

4. Have the authors made all data underlying the findings in their manuscript fully available?

The PLOS Data policy requires authors to make all data underlying the findings described in their manuscript fully available without restriction, with rare exception (please refer to the Data Availability Statement in the manuscript PDF file). The data should be provided as part of the manuscript or its supporting information, or deposited to a public repository. For example, in addition to summary statistics, the data points behind means, medians and variance measures should be available. If there are restrictions on publicly sharing data—e.g. participant privacy or use of data from a third party—those must be specified.

Reviewer #5: Yes

**********

5. Is the manuscript presented in an intelligible fashion and written in standard English?

PLOS ONE does not copyedit accepted manuscripts, so the language in submitted articles must be clear, correct, and unambiguous. Any typographical or grammatical errors should be corrected at revision, so please note any specific errors here.

Reviewer #5: Yes

**********

6. Review Comments to the Author

Please use the space provided to explain your answers to the questions above. You may also include additional comments for the author, including concerns about dual publication, research ethics, or publication ethics. (Please upload your review as an attachment if it exceeds 20,000 characters)

Reviewer #5: I have no further comments, other than the "benefitted" phrase was used in the abstract and that could be replaced with the better phrase.

**********

7. PLOS authors have the option to publish the peer review history of their article (what does this mean?). If published, this will include your full peer review and any attached files.

If you choose “no”, your identity will remain anonymous but your review may still be made public.

**Do you want your identity to be public for this peer review?**  For information about this choice, including consent withdrawal, please see our Privacy Policy.

Reviewer #5: No

**********

# Editor report: Acceptance letter

**DOI:** 10.1371/journal.pone.0317638.r010

**Authors:** Marcelo Hermes-Lima, Marcelo Hermes-Lima, Yuqing Li, Yuqing Li, Yuqing Li

PONE-D-25-00224R4

PLOS One

Dear Dr. Navarro,

I'm pleased to inform you that your manuscript has been deemed suitable for publication in PLOS One. Congratulations! Your manuscript is now being handed over to our production team.

At this stage, our production department will prepare your paper for publication. This includes ensuring the following:

* All references, tables, and figures are properly cited

* All relevant supporting information is included in the manuscript submission,

* There are no issues that prevent the paper from being properly typeset

You will receive further instructions from the production team, including instructions on how to review your proof when it is ready. Please keep in mind that we are working through a large volume of accepted articles, so please give us a few days to review your paper and let you know the next and final steps.

Lastly, if your institution or institutions have a press office, please let them know about your upcoming paper now to help maximize its impact. If they'll be preparing press materials, please inform our press team within the next 48 hours. Your manuscript will remain under strict press embargo until 2 pm Eastern Time on the date of publication. For more information, please contact onepress@plos.org.

You will receive an invoice from PLOS for your publication fee after your manuscript has reached the completed accept phase. If you receive an email requesting payment before acceptance or for any other service, this may be a phishing scheme. Learn how to identify phishing emails and protect your accounts at https://explore.plos.org/phishing.

If we can help with anything else, please email us at customercare@plos.org.

Thank you for submitting your work to PLOS ONE and supporting open access.

Kind regards,

PLOS ONE Editorial Office Staff

on behalf of

Dr. Yuqing Li

Academic Editor

PLOS One
